# Impact of Hfq on Global Gene Expression and Intracellular Survival in *Brucella melitensis*

**DOI:** 10.1371/journal.pone.0071933

**Published:** 2013-08-19

**Authors:** Mingquan Cui, Tongkun Wang, Jie Xu, Yuehua Ke, Xinying Du, Xitong Yuan, Zhoujia Wang, Chunli Gong, Yubin Zhuang, Shuangshuang Lei, Xiao Su, Xuesong Wang, Liuyu Huang, Zhijun Zhong, Guangneng Peng, Jing Yuan, Zeliang Chen, Yufei Wang

**Affiliations:** 1 Department of Infectious Disease Control, Institute of Disease Control and Prevention, Academy of Military Medical Sciences, Beijing, China; 2 College of Veterinary Medicine, Sichuan Agricultural University, Ya’an, China; 3 State Key Laboratory of Microbial Resources, Institute of Microbiology, Chinese Academy of Sciences, Beijing, China; University of Louisville, United States of America

## Abstract

*Brucella melitensis* is a facultative intracellular bacterium that replicates within macrophages. The ability of brucellae to survive and multiply in the hostile environment of host macrophages is essential to its virulence. The RNA-binding protein Hfq is a global regulator that is involved in stress resistance and pathogenicity. Here we demonstrate that Hfq is essential for stress adaptation and intracellular survival in *B. melitensis*. A *B. melitensis hfq* deletion mutant exhibits reduced survival under environmental stresses and is attenuated in cultured macrophages and mice. Microarray-based transcriptome analyses revealed that 359 genes involved in numerous cellular processes were dysregulated in the *hfq* mutant. From these same samples the proteins were also prepared for proteomic analysis to directly identify Hfq-regulated proteins. Fifty-five proteins with significantly affected expression were identified in the *hfq* mutant. Our results demonstrate that Hfq regulates many genes and/or proteins involved in metabolism, virulence, and stress responses, including those potentially involved in the adaptation of *Brucella* to the oxidative, acid, heat stress, and antibacterial peptides encountered within the host. The dysregulation of such genes and/or proteins could contribute to the attenuated *hfq* mutant phenotype. These findings highlight the involvement of Hfq as a key regulator of *Brucella* gene expression and facilitate our understanding of the role of Hfq in environmental stress adaptation and intracellular survival of *B. melitensis*.

## Introduction

*Brucella spp.* are gram-negative intracellular pathogens that belong to the α-2 subclass of proteobacteria, which live in close association with eukaryotic hosts [1]. Bacteria of the genus *Brucella* are the etiological agents of brucellosis, a worldwide zoonotic disease that affects a broad range of mammals and causes great economic losses [2], [3]. Human brucellosis is a chronic and debilitating febrile illness commonly referred to as Malta fever or undulant fever. The ability of brucellae to establish and maintain chronic infections depends upon its ability to survive and replicate within host phagocytes [4]. The intracellular environment of phagocytic cells is potentially hostile to microorganisms; however, intracellular pathogen can adapt to changes in their environment, avoiding degradation by host cell defense systems through the coordinated regulation of gene expression.

Hfq is a bacterial Sm-like protein that acts as a post-transcriptional regulator of global gene expression [5], [6]. The Hfq protein is highly conserved among bacteria, which was originally identified in *Escherichia coli* as a host factor essential for the replication of Qβ RNA bacteriophage [7]. Approximately a decade ago, Hfq was revealed to be a key factor in regulation the RNA-RNA interactions between small regulatory RNAs (sRNAs) and their mRNA targets [5], [6], [8]. Additionally, Hfq is required for the expression of some target genes in the absence of sRNA, by modulating the half-life of mRNAs directly or allowing the polyadenylation of mRNAs [5], [9]. Hfq has been shown to be involved in a wide range of cellular processes and pathways [10]. Consequently, in many bacteria, *hfq* inactivation results in a pleiotropic phenotype that includes alterations in the growth rate, an impaired resistance to various environmental stresses, and an altered virulence [11]–[17]. Robertson and Roop demonstrated that a *Brucella abortus* Δhfq mutant was defective in its ability to invade and survive inside animal cells and was more sensitive to stress environments, thus indicating the contribution of Hfq to the intracellular survival of *B. abortus* [11].

Although a few studies have reported that Hfq coordinates the expression of some *Brucella abortus* genes that are involved in adaptive responses to stress conditions and virulence, including those that encode the superoxide dismutase SodC [18], the acid resistance protein HdeA [19], the type IV secretion system VirB, and the LuxR-type transcriptional regulator BabR [20]. However, the full repertoire of Hfq-dependent genes has not been elucidated in *Brucella*. In this study, we performed sample-matched global transcriptome and proteome analyses to determine the global Hfq-dependent changes in gene expression and protein accumulation. The data demonstrated that 11% of the *B. melitensis* genes were either directly or indirectly affected when the *hfq* gene was deleted, and this deletion was accompanied by attenuated virulence and altered physiological characteristics. The results will help us to understand how Hfq controls *B. melitensis* gene expression and the role that may play in the environmental adaptation and intracellular survival of *B. melitensis*.

## Materials and Methods

### Ethics Statement

All animal experiments were performed in strict accordance with Experimental Animal Regulation Ordinances defined by China National Science and Technology Commission, and the protocol was approved by Beijing Institute of Disease Control and Prevention animal ethics committee. Animals are provided with humane care and healthful conditions during their stay in the facility. All individuals who use animals receive instruction in experimental methods and in the care, maintenance and handling of mice, and are under the committee’s supervision.

### Bacterial Strains and Plasmids

*B. melitensis* 16 M was routinely cultured in rich medium Tryptic Soy Broth (TSB) or in minimal medium GEM7.0 (MgSO_4_.7H_2_O 0.2 g/L, Citric acid•H_2_O 2.0 g/L, K_2_HPO_4_ 10.0 g/L, NaNH_4_HPO_4_.4H_2_O 3.5 g/L, Glucose 20 g/L, pH 7.0) at 37°C. *E. coli* strain DH5α was grown on Luria–Bertani (LB) medium. Plasmid pBBR1MCS-5, a broad host range plasmid capable of replicating in *Brucella*, was kindly provided by Professor Kenneth M. Peterson [21].

### Generation of Mutant and Complementary Strain

The Hfq deletion strain 16 MΔhfq was generated by resistance gene replacement as described previously [22]. Approximately 500 bp sequences of the upstream and downstream regions of *hfq* coding region were assembled in pUC19K [22] to generate suicide plasmid pUC19K-hfq. Competent 16 M was electroporated with pUC19K-hfq and potential *hfq* deletion mutant 16 MΔhfq was isolated by its amp^S^ kan^R^ phenotype. The deletion mutant was further confirmed by PCR amplification with primer pUC19K-F and hfq-I-R, which located in kamamycin gene and downstream of homologous arm of *hfq* respectively. PCR products were sequenced to confirm the sequence. The deletion mutant was further confirmed by RT-PCR.

The complementary strain was constructed as follows. The wild-type *hfq* locus was amplified using primers Hfq-N-F and Hfq-C-R, genomic DNA from *B. melitensis* 16 M as a template. Then, the DNA fragments were treated with *Kpn*I and *Pst*I, and ligated into *Kpn*I/*Pst*I-digested pBBR1MCS5, a plasmid that could replicate in *Brucella*. The resulting plasmid pBBR1-hfq was electroporated into 16 M△hfq, resulting in the complementary strain 16 M△hfq-C. The transcription restoration of *hfq* in the complementary strain was further confirmed by RT-PCR.

### Growth Curve, in vitro Environmental Stress and Virulence Studies of *hfq* Deletion Mutant

For growth curve analysis, *B. melitensis* 16 M, 16 MΔhfq and 16 MΔhfq-C were cultured in TSB for 24 h, and then diluted with TSB to an OD600 of 0.05 and cultured in a rotary shaker (250 rpm) at 37°C. Aliquots of the cultures were taken at an interval of 2 h and cell density (OD600) was recorded.

The susceptibilities of *B. melitensis* 16 M, 16 MΔhfq and 16 MΔhfq-C to the various in vitro environmental stress conditions were determined as follows. *B. melitensis* strains inoculated into TSB medium were grown to the early logarithmic phase (OD600 = 0.6) at 37°C. To determine the effect of high-salinity or high-osmolarity stress on *B. melitensis*, the log-phase cells were incubated at 37°C for 20 min in the presence of NaCl (1.5 M). For acidification stress, the cells were incubated at 37°C for 15 min in TSB medium at pH 3.0. For oxidative stress, the cells were incubated at 37°C for 40 min in the presence of 440 mM H_2_O_2_. For heat shock, the cells were transferred to pre-warmed 50°C tubes and incubated at 50°C for 60 min. For antibacterial peptides, the cells were incubated at 37°C for 60 min in the presence of 500 µg/ml polymyxin B. After the treatment, cells were diluted and plated on TSA plates to determine the number of viable bacteria. All the results represent the means from at least three separate experiments.

Murine macrophage-like RAW264.7 were used to assess survival capability of 16 MΔhfq mutant, 16 MΔhfq-C and their wild type strain 16 M. Briefly, mono-layers of macrophages of 5×10^5^ cells/well were cultured in 24-well plate for 16 h at 37°C, infected with *Brucella* at a MOI of 50. At 45 min post-infection, the cells were washed twice with PBS and then incubated with 50 µg/ml of gentamicin for 60 min to kill extra-cellular bacteria. Then, the cultures were replaced with DMEM with 25 µg/ml of ampicilin. At 0, 8, 24, and 48 hours post the infection, the supernatant was discarded and cells were lysed, and the live bacteria were enumerated by plating on TSA plates. All assays were performed in triplicate and repeated at least three times.

For mouse virulence assay, 6- to 8-week-old female BALB/c mice (five per *B. melitensis* strain per time point) were infected intraperitoneally with 1×10^7^ CFU of each *Brucella* strain in sterile PBS. 7 and 28 days post the infection, mice were sacrificed by cervical dislocation and spleens were removed aseptically and homogenized with PBS containing 0.1% Triton X-100. The homogenates were serially diluted and plated on TSA, and the CFU were counted after 5 days of incubation at 37°C.

### Determination of in vitro Induction Conditions for *hfq*

*B. melitensis* 16 M was grown in TSB to the stationary phase at 37°C and then subjected to TSB4.0 (acid shock), GEM7.0 (limited nutrition), GEM4.0 (limited nutrition and acid shock), TSB with 1.5 mM H_2_O_2_ (oxidative stress), 42°C (heat shock), TSB7.0 (control) for 30 min. Then, the transcription of *hfq* under these stresses was compared by quantitative RT-PCR.

### RNA Preparation and Microarray Analysis

16 M and 16 MΔhfq strains were grown in TSB at 37°C to the stationary phase, and then transferred to the stress condition where *hfq* was greatly activated. Total RNA was extracted from liquid cultures of *B. melitensis* using Trizol reagent (Invitrogen) as recommended by the manufacturer. RNA samples were then treated with DNAse I (Promega) to remove any contaminating genomic DNA. RNA quantity and quality were assessed using ND-1000 Spectrophotometer Nanodrop (Technologies) and agarose gel electrophoresis.

Twenty micrograms of RNA from 16 M or 16 MΔhfq were used to synthesize cDNA in the presence of aminoallyl-dUTP, genome directed primers (GDPs) and random hexamer primers with the Superscript II system (Invitrogen). The aminoallyl modified cDNA was then labeled by Cy5 or Cy3 monofunctional dye (Amersham) as described previously. The Cy3 and Cy5 reaction products were mixture, and the unincorporated dye was removed using QiaQuick columns (Qiagen). The purified probes were dried in SpeedVac. Glass slides spotted with PCR amplicons representing about 99% of non-redundant annotated ORFs of *B. melitensis* 16 M and *B. abortus* 9–941 were used for probe hybridization [23]. The DNA microarrays were cross-linked using a UV Stratalinker (Hoefer). NaBH_4_ was used to block the free aldehyde groups on the slide surface. The slides were incubated in a prehybridization buffer (5×SSC, 0.1% SDS, and 0.1% BSA) and then washed and blown to dry by hot air. The Cy3/Cy5 differentially labeled cDNA samples were resuspended in hybridization solution (50% deionized formamide, 5×SSC, 0.1% SDS, 5×Denhardt’s solution, and 0.5µg/µl of sheared salmon sperm DNA), and hybridized with the slides at 42°C for 18–20 h. Then, the slides were washed and scanned using a GenePix Personal 4100 A microarray scanner (Axon Instruments).

The scanning images were processed and the data were further analyzed by using GenePix Pro 4.1 software (Axon Instruments) in combination with Microsoft Excel software. Spots were analyzed by adaptive quantitation, and the local background was subsequently subtracted. Spots with background-corrected signal intensity (median) in both channels lower than 2-fold of background intensity (median) were rejected from further analysis, and then the remaining data points were normalized by total intensity normalization methods. The normalized log_2_ ratio of test/reference signal for each spot was recorded. The averaged log_2_ ratio for each gene with at least four data points was finally calculated. All microarray data reported in the study is described in accordance with MIAME guidelines and has been deposited in NCBI’s Gene Expression Omnibus (GSE 46418). Significant changes of gene expression were identified with Significance Analysis of Microarrays (SAM) software.

### Two-dimensional (2-DE) Gel Electrophoresis and MALDI-TOF/TOF MS Analysis

16 M and 16 MΔhfq strains were grown in TSB at 37°C to the stationary phase, and then transferred to the stress condition where *hfq* was greatly activated. Bacterial cells were harvested and centrifuged at 8,000 rpm for 5 m in at 4°C. The cells pellet were resuspended in 5 mL of lysis buffer (7 M urea, 2 M thiourea, 4% (w/v) CHAPS and 50 mM DTT) containing complete protease inhibitors (Roche Diagnostics). The cells were sonicated for 5 minutes (cycles of 2 s of sonication followed by a 3 s rest) on ice with a Sonifier 750 (Branson Ultrasonics Corp) set at 30% duty cycle. After adding 2.5 mg of RNase A (Promega) and 100 unit of RQ1 DNase (Promega), the cell lysate was incubated for 1 hour at 15°C to solubilize proteins, and then centrifuge for 40 min at 20,000×g to pellet the insoluble components. The supernatant was collected, and protein concentration was measured by the PlusOne 2-D Quant Kit (GE Healthcare Life Sciences).

The 2-DE procedure was conducted according to a previously published protocol [24]. Briefly, 1 mg proteins extracted from the related strains were separated by 2-DE using a linear pH 4–7 IPG strips (18 cm) and SDS-PAGE. Preparative gels used for identification of proteins were stained with Coomassie Brilliant Blue G-250 (Amresco). Gels were scanned and images were analyzed with ImageMaster 2-D Elite version 5.0 software. The relative volume of each spot was determined from the spot intensity in pixel units and normalized to the sum of the intensities of all the spots in the gel. Proteins were considered differentially expressed if their relative volume deviated more than 2 folds. Each experiment was performed at least three times.

The protein spots of interest were cut out of the gel and destained with 50 µl of 25 mM ammonium bicarbonate in 50% acetonitrile (ACN) for 30 min at room temperature for three times. The destained gel pieces were completely dried in a Speedvac vacuum concentrator (Savant Instruments). The samples were resolubilized in 3 µl of 25 mM ammonium bicarbonate containing 10 ng of trypsin at 4°C for 1 h. After 13 h of incubation at 37°C, the gels were dried in a high vacuum centrifuge to evaporate solvent. 8 µl of 5% trifluoroacetic acid (TFA) was added to the gel spots and incubated at 37°C for 1 h. The supernatant was transferred into a new microtube containing the supernatant from the TFA extraction. Finally, 8 µl of 100% ACN was used for extraction of hydrophobic peptides. All of the supernatants were combined and dried in the SpeedVac vacuum concentrator, and resolubilized with 2 µl of 0.5% TFA. Then, they were analyzed by MALDI-TOF/TOF MS measurements as described in a previous study [24].

The MS/MS results were searched against the NCBInr database using the MASCOT search program (www.matrixscience.com). To eliminate redundancy resulting from multiple members of the same protein family, the proteins of strain *B. melitensis* 16 M were chosen for the further analyses. The search parameters are as following: trypsin digestion with two missed cleavage; carbamidomethyl modification of cysteine and oxidation of methionine as variable modifications; peptide tolerance maximum, ±0.3 Da; MS/MS tolerance maximum, ±0.5 Da; peptide charge, +1; monoisotopic mass. We did not identify any protein with more than one name and one accession number in the above databases. Thresholds refer to significant P values (p<0.05) of Mascot results. Scores greater than 84 are significant (p<0.05) for Peptide Mass Fingerprinting (PMF) search. Ion scores greater than 46 are significant (p<0.05) for a local MS/MS search.

### Reverse Transcriptase PCR (RT-PCR)

RT-PCR were performed in 25 µl volumes containing 12.5 µl of 2×SYBR Green I Master Mix (Takara Biochemicals), 100 nM each primer, and 1 µl of cDNA sample. Thermocycling conditions were as follows: 10 min at 95°C for pre-incubation, and then 45 cycles of amplification (95°C for 30 s, 60°C for 30 s, and 72°C for 30 s). The primers used for RT-PCR are listed in Table S1. All primer sets showed standard curves with R^2^ values of >0.980, 90 to 110% reaction efficiencies, and only one peak in dissociation curves. Relative transcriptional level was determined by the methods of 2^−△△Ct^ as described previously [25]: relative fold change (treatment/control) = 2^−△△Ct^, where △Ct (gene of interest) = Ct (gene of interest)−Ct (reference gene of the same sample) and △△Ct (gene of interest) = △Ct (treatment)−△Ct (control). The level of 16S rRNA was used as a reference gene to normalize the expression data for target gene.

### Statistical Analysis

Bacteria survival under in vitro stresses was expressed as the mean percent survival compared to untreated controls ± the standard deviation (SD). Statistical analysis was performed with Student’s unpaired t test. Bacteria survival in macrophage and in mice was expressed as the mean log_10_CFU ± SD. The differences between groups were analyzed by ANOVA followed by Tukey’s honestly significant difference posttest comparing all groups to one another. For qRT-PCR experiments, significance was calculated by the Wilcoxon signed-rank test. In all cases, a *P* value of less than 0.05 was considered significant.

## Results and Discussion

### Construction and Growth Characteristics of the *B. melitensis* Δhfq Mutant

**Figure 1:**
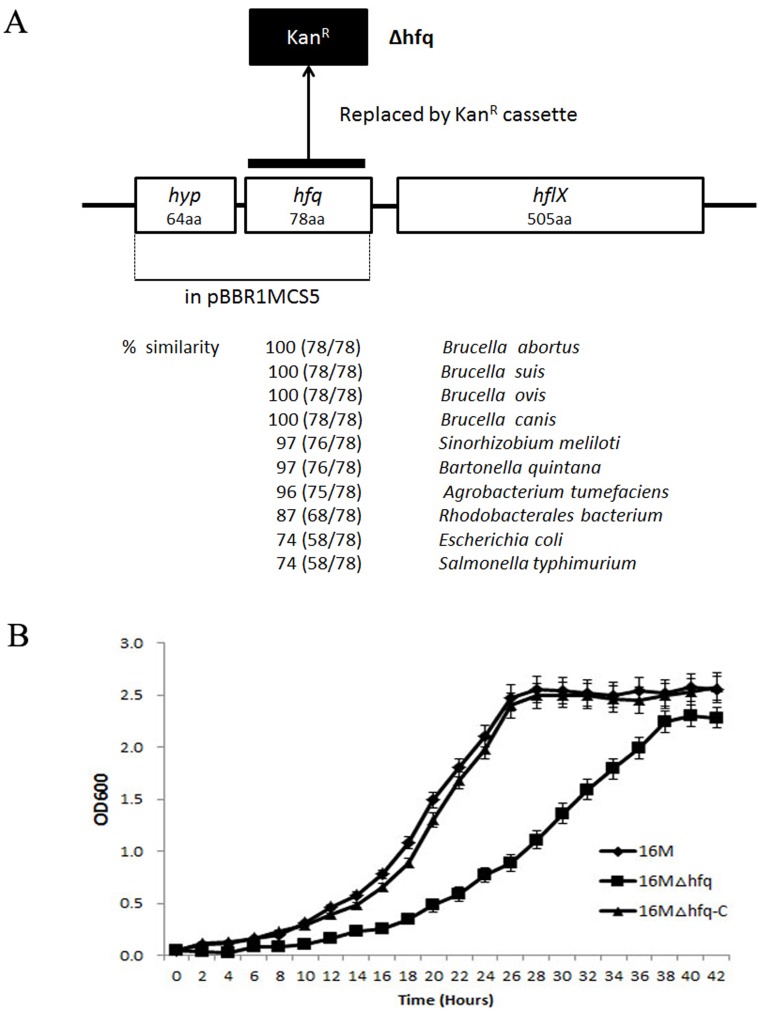
Details of the *B. melitensis hfq* mutants and their growth characteristics. A. Structure of the *hfq* locus on *B. melitensis* 16 M chromosome I. In the Δhfq, the coding region of *hfq* was deleted and replaced by a kanamycin resistance cassette. The coding region of *hfq* together with its native promoter cloned to pBBR1MCS5 yielding the complementation plasmid pBBR1-hfq. The percentage similarities were obtained using pairwise BLAST analyses that compared *B. melitensis* Hfq protein sequence with those of other *Brucella spp*., alpha-proteobacteria, *Escherichia coli*, and *Salmonella typhimurium*. B. Growth characteristics of *B. melitensis* Δhfq strain. *B. melitensis* wild-type, 16 MΔhfq, and 16 MΔhfq-C strain were cultured in TSB (pH7.0) at 37°C, and the OD600 was measured at 2 h intervals. Each graph represents the mean of three independent biological replicates grown on three different days. The error bars represent the standard deviation (SD) of the optical density at each time point.

The *hfq* gene (BMEI0872) is located in clockwise orientation at bps 900419–900655 in the genome of *B. melitensis* 16 M chromosome I and it is flanked by the *hflX* gene, encoding the gtp-binding protein, and a *hyp* gene, encoding a small hypothetical protein (Fig. 1A). Sequence analysis revealed the ORF consists of 234 nucleotides that could encode a protein of 78 amino acids. The protein sequence of Hfq is highly conserved among different other *Brucella* spp. It also showed high protein sequence similarity (97–87%) with other alpha-proteobacteria. Hfq is essential for virulence in a variety of pathogenic bacteria, including *B. abortus*. To further address the role of Hfq in the intracellular survival of *B. melitensis*, we constructed the *hfq* mutant using a gene replacement strategy. RT-PCR analysis was performed to ensure that the mutant strain did not express *hfq*. Then, the Hfq loci were reconstructed in the *hfq* deletion mutant to generate a plasmid-based complementary clone of *hfq*. Transcription of *hfq* was restored in the complemented strain, and *hfq* was noticeably overproduced in this strain compared to wild type, likely due to the multicopy nature of the plasmid (data not shown). The effects of Hfq on *B. melitensis* growth in nutrient-replete (TSB7.0) or nutrient-limiting (GEM7.0) media were then examined. When cultured in rich TSB media, the 16 MΔhfq mutant showed a longer lag phase and reached the stationary phase at a lower optical density compared with the wild-type strain (Fig. 1B). Moreover, the Hfq-deficient strain also exhibited a decreased growth rate in minimal media (GEM7.0) (data not shown). Genetic complementation of *hfq* restored 16 MΔhfq growth in both TSB7.0 and GEM7.0. These results suggest that Hfq is involved in broad cellular functions, including growth rate regulation.

### Reduced Stress Tolerance and Intracellular Survival of the *B. melitensis* Δhfq Mutant

**Figure 2:**
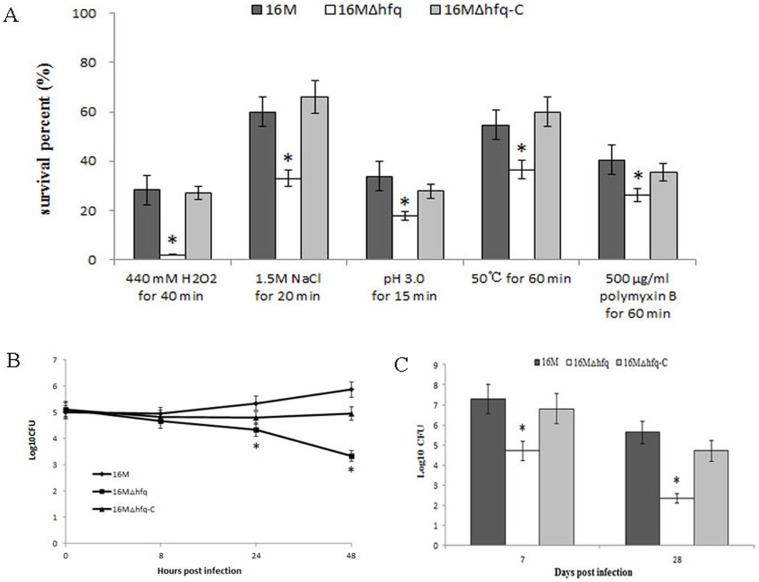
Survival of the *B. melitensis* Δhfq mutant strain in in vitro environmental stress conditions, macrophages, and mice. A. In vitro stress resistance analysis of *B. melitensis* Δhfq mutant strain. 16 M, 16 MΔhfq, and 16 MΔhfq-C were grown in TSB to the early logarithmic phase and then subjected to different stress conditions as described in the text. After the treatments, the surviving bacteria were enumerated by plating serial dilutions onto TSA plates. Bars represent mean percent survival compared to untreated controls, and error bars represent standard errors of percent survival from 3 replicates. Significant differences between the mutant and parent strain are indicated as follows: *, P<0.001. B. Survival capacity of *B. melitensis* Δhfq mutant strain in macrophage cells. RAW264.7 cells were infected with strains 16 M, 16 MΔhfq, or 16 MΔhfq-C at a MOI of 50∶1. Three wells were evaluated at each time point for every strain tested, and the colony forming units were determined by serial dilution and plating on TSA. The data was expressed as the mean log_10_CFU ± SD (n = 3). Significant differences between the mutant and parent strain are indicated as follows: *, P<0.001. C. Survival capacity of *B. melitensis* Δhfq mutant strain in BALB/c mice. Groups of five BALB/c mice were infected intraperitoneally with 1×10^7^ CFU of 16 M, 16 MΔhfq, or 16 MΔhfq-C. At 7 and 28 days post-infection, the spleens were aseptically removed and the colony forming units were determined by plating serial dilutions on TSA plates. The data was expressed as the mean log_10_CFU ± SD (n = 5). Significant differences between the mutant and parent strain are indicated as follows: *, P<0.001.

As intracellular bacterial pathogens, *Brucella* species can survive and replicate in host phagocytes, where they likely encounter different stresses such as oxidative stress, low pH, limited nutrition, high osmolarity, and antibacterial peptides [26]. A previous report showed that *B. abortus hfq* mutants exhibited increased sensitivity to H_2_O_2_ and decreased survival under acidic conditions [11]. To further determine the role of Hfq in *B. melitensis* stress tolerance, the susceptibilities of a wild-type strain, the Δhfq mutant, and 16 M△hfq-C to various stress conditions was compared. As shown in Fig. 2A, when compared with the wild-type strain, the *hfq* gene deletion in *B. melitensis* caused the survival percentage to decrease by 30∼50% upon exposure to high osmolarity, acidic pH, heat shock, and antibacterial peptide. Under oxidative stress, the survival percentage of 16 MΔhfq decreased even further (90%). The decreased survival of 16 MΔhfq was recovered in the complementary strain 16 M△hfq-C, indicating that the reduced survival was dependent on the inactivation of Hfq. These data demonstrated that Hfq plays an important role in the resistance of *B. melitensis* to a wide range of stresses, including those relevant to host environments.

Since the ability to replicate within macrophages is essential to *Brucella* virulence, we examined the intracellular survival and replication of the *B. melitensis* Δhfq mutant strain in macrophages. RAW264.7 macrophages were infected with 16 M, 16 MΔhfq, or 16 M△hfq-C at a multiplicity of infection of 50, and the surviving bacteria were enumerated. At 0 h, equivalent bacteria loads were observed in the RAW264.7 cells, indicating that the loss of *hfq* did not affect the invasion of *B. melitensis* into the macrophages. By 8 h post-infection, there were no significant differences in the number of surviving bacteria in the infected macrophages. However, at 24 h post-infection, there was a 1.0-log decrease (P<0.001) in the bacterial number of 16 MΔhfq compared to that of 16 M; this decrease progressed to a 2.5-log difference at 48 h post-infection (Fig. 2B). These results indicate that Hfq is required for prolonged survival in the intracellular environment of host macrophages, which is consistent with the earlier report [11].

To further address the role of Hfq in *B. melitensis* pathogenesis, BALB/c mice were infected intraperitoneally with the *B. melitensis* wild-type 16 M, 16 MΔhfq, or 16 M△hfq-C, and spleen colonization by the brucellae was assessed at 7 and 28 days after infection. Hfq mutation significantly reduced the *B. melitensis* counts in the spleens of the infected animals, and the reduced virulence was recovered in the complementary strain 16 M△hfq-C (Fig. 2C). Taken together, these results suggest that Hfq is involved in the intracellular survival and pathogenicity of *B. melitensis*, similar to previous reports of other pathogens [11]–[17]. When our manuscript was revised, a study also reported that *B. melitensis* Δhfq mutants showed reduced survival in macrophages and mice [27], again confirming the role of Hfq in *B. melitensis* virulence.

### Global Effects of Hfq on the *B. melitensis* Transcriptome and Proteome

**Figure 3:**
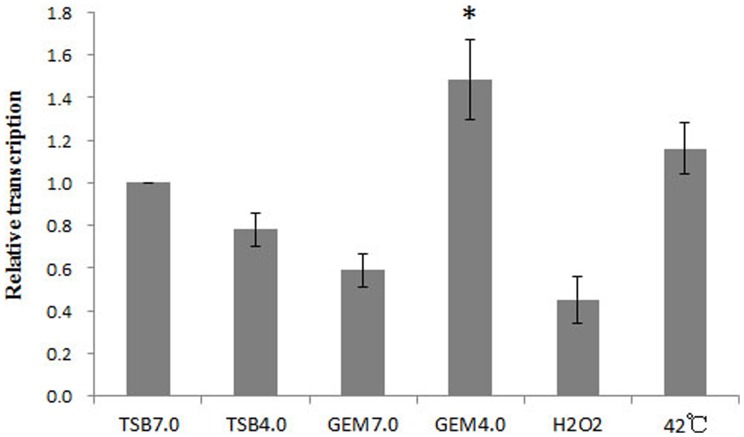
Determination of the in vitro induction conditions for *hfq*. 16 M was firstly cultured in TSB (pH7.0) to the stationary phase and then subjected to different stresses. RNA was isolated and transcription of *hfq* was quantified by qRT-PCR. Significant differences between the acidified minimum medium (GEM 4.0) and other in vitro stresses are indicated as follows: *, P<0.05.

Hfq is highly activated during host infections but can also be activated under a variety of in vitro conditions. To determine genes regulated by Hfq, the transcriptome and proteome of the wild type and mutant strains were compared under in vitro condition in which the transcription of *hfq* was highly induced. To identify such conditions, *B. melitensis* 16 M was grown to the stationary phase and subjected to several in vitro conditions that simulated conditions encountered in hosts’ phagocytes; the relative *hfq* transcript levels under each condition were then compared using quantitative RT-PCR (qRT-PCR). The results indicated that the transcriptional level of *hfq* in acidified minimum medium (GEM 4.0) was significantly higher than those induced by other in vitro stresses (Fig. 3). Thus, the transcriptome and proteome of the wild type and Δhfq mutant strains were compared under GEM 4.0 with the reasoning that large differences in the expression levels might be observed.

**Figure 4:**
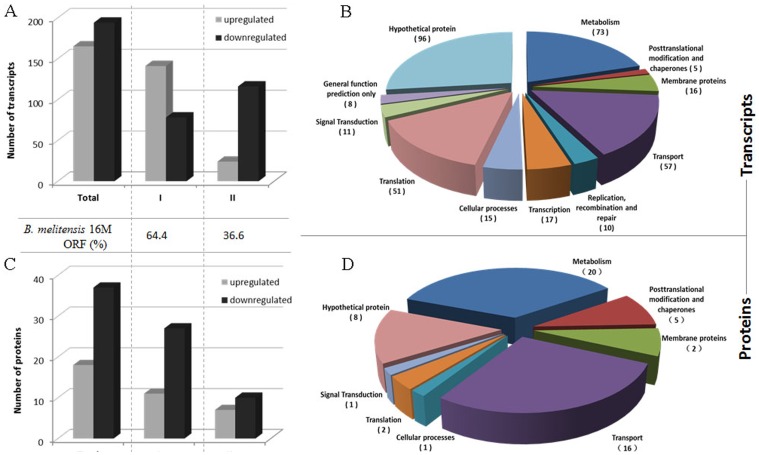
Differentially expressed transcripts (upper graphs) and proteins (lower graphs) in the *B. melitensis* Δhfq mutant strain. Histograms show the number of differentially expressed genes and their distributions in the *B. melitensis* chromosome. The functional categories according to the *B. melitensis* 16 M genome sequence annotation and the KEGG database is shown to the right in circle charts. The number of genes belonging to each category are shown in brackets.

Hfq-dependent changes in transcript abundance were first investigated by comparing the expression profiles of the wild-type 16 M and 16 MΔhfq strains grown in GEM 4.0. Of the predicted 3,198 ORFs in the *B. melitensis* genome, a total of 359 genes (approximately 11%) showed a >2.0-fold change in transcript abundance in Δhfq when compared with 16 M. Among the 359 Hfq-dependent genes, 194 were found to be down-regulated and 165 were found to be up-regulated in the 16 MΔhfq strain. According to the *B. melitensis* 16 M genome sequence annotation (NC_003317 and NC_003318) and the KEGG database, the 359 Hfq-dependent genes belonged to 11 functional categories (Fig. 4B and Table S2), with genes of unknown or unclassified function (104/359) representing the largest functional category as expected. Among the remaining genes with known functional homology, 51% (130/255) were predicted to encode proteins and enzymes for transport and metabolism, and these genes constituted the largest class identified in the *B. melitensis* 16 M Hfq regulon. The remaining genes were related to translation, transcription, membrane function, cellular processes, signal transduction, recombination and repair, and posttranslational modification.

In order to investigate the effects of Hfq on global protein expression in *B. melitensis*, the proteomic profiles of the wild-type strain 16 M and its Δhfq mutant grown in GEM 4.0 were compared by 2-D gel electrophoresis. The typical proteome gel maps of 16 M and 16 MΔhfq were presented in Figure S1. The relative volumes of protein expression were determined by imaging analysis, and 52 protein spots were found to be upregulated and 76 were found to be downregulated in the *hfq* mutant when compared to the wild-type strain. The differentially expressed protein spots were picked from the preparative gels for MALDI-TOF mass spectrometry identification. Among the spots analyzed by MALDI-TOF/TOF MS, only the 61 spots that were matched to a single protein in *B. melitensis* were retained. The 61 differentially expressed protein spots represented 55 proteins, of which, 18 were over-represented and 37 under-represented in the Δhfq mutant strain (Table S3). The functions of the proteins with altered abundances can be subdivided into various groups (Fig. 4D), including transport and metabolism (36/55), outer membrane proteins (2/55), posttranslational modification (5/55), translation (2/55), cellular processes (1/55), signal transduction (1/55), and hypothetical proteins (8/55). Roop et al. investigated the *B. abortus* Δhfq mutant strain proteome by using 2D gel analysis and identified 6 differentially expressed proteins [28]. Among these 6 proteins, only SodC (BMEII0581) was identified in our study. This discrepancy could be attributed to the different *Brucella* species and different growth conditions used.

A comparison of the transcriptomic and proteomic profiles described in this study revealed 12 overlapping genes which had been identified as differentially expressed in the *hfq* mutant and wild-type strain in both analyses (Table S3). Ten of these 12 genes were involved in transport and metabolism. The lack of correlation between the transcriptomes and proteomes could be due to the role of Hfq as a post-transcriptional regulator that decouples transcription and translation. Similar results were also observed for *Salmonella* and *Sinorhizobium meliloti* [29], [30].

### Effect of Hfq on Transporters and Metabolic Genes

The transcriptomic profiling data suggested that 73 genes predicted to be involved in cellular metabolism were differentially regulated in the Δhfq deletion mutant when grown in GEM 4.0 conditions, including genes involved in the metabolism of amino acids (14), carbohydrates (14), energy (15), inorganic ions (4), cofactors and vitamins (6), nucleotides (12), lipids (2), xenobiotics (2), and glycans (2). Additionally, in the Δhfq mutant, transcripts that corresponded to 57 transporter-related genes were differentially expressed, including the different components of the ABC-type transporters (i.e., the periplasmic solute binding protein, the permease, or the ATP-binding protein). These transporters are mainly involved in the uptake of amino acids, peptides, inorganic ions and sugars. The proteomic analysis results also indicated that transport and metabolism represented the dominant class of proteins affected by the *hfq* mutation. Among the 55 differentially expressed proteins, 36 were associated with transportation and metabolism. Thus, both approaches support the general conclusion that Hfq influences the cellular metabolic activity of *B. melitensis*. This might be the molecular basis of the growth deficiency observed in the *hfq* mutant (Fig. 1B).

Compared to the levels for the *B. melitensis* 16 M WT strain, 5 proteins and 14 transcripts associated with central carbon metabolism were all down-regulated in the *hfq* mutant. Moreover, most of the transporters involved in the uptake of different sugars (BMEI1716, BMEII0086, BMEII0114, BMEII0360, BMEII0361, BMEII0435, BMEII0590, and BMEII1053) were also less abundant in the mutant, indicating a reduced efficiency in the import of primary carbon substrates. Additionally, the lack of Hfq led to the dysregulation of transporters associated with inorganic ion uptake. The copper- (BMEII0972) and taurine- (BMEII0108, *tauB*) binding proteins were down-regulated, whereas the manganese- (BMEI0569, *mntH*) and molybdate- (BMEII0005) binding proteins were up-regulated. As for the transporters involved in iron ion uptake, BMEII0535 and BMEII1120 were over-represented and BMEII0584 and BMEII0604 were under-represented in the Δhfq mutant strain, indicating that Hfq may involve in multiple iron metabolism pathways in *B. melitensis*. The involvement of Hfq in the regulation of iron and manganese uptake proteins has also been documented in other organisms [31], [32].

### Many Stress- or Virulence-related Genes Affected by Hfq

#### Virulence-related genes

The type IV secretion system encoded by the *virB* operon is essential for the intracellular survival and full virulence of *Brucella* [25], [33]. Transcriptomic data showed that the lack of Hfq led to a reduction in the abundance of the *virB* transcript, which was consistent with the report by Caswell et al [20]. MucR is a Ros-type transcriptional regulator that has been linked to virulence in *Brucella* [34]. The *B. abortus* ΔmucR mutant strain exhibited a pronounced growth defect during in vitro cultivation and was attenuated in cultured macrophages and in mice. MntH is an H^+^-dependent manganese transporters and it is the sole high-affinity manganese transporter in *Brucella* [35]. The *B. abortus mntH* mutant exhibited an increased susceptibility to oxidative killing in vitro. Moreover, the ΔmntH mutant also exhibited extreme attenuation in both cultured murine macrophages and mice, indicating that MntH is required for *B. abortus* virulence. Our results showed that *hfq* deletion in *B. melitensis* caused the dysregulation of *mucR* and *mntH*. Additionally, Hfq is also involved in regulating the VjbR quorum sensing transcriptional regulator that has been shown to be essential for *Brucella* virulence [36].

### Outer Membrane Proteins

Outer membrane proteins (OMPs) are essential for the maintenance of membrane integrity and selective permeability [37]. Additionally, OMPs are often regulated by environmental signals and play important roles in bacterial pathogenesis by enhancing adaptability to various environments [38], [39]. Previous studies indicated that the lack of *hfq* could result in OMPs overproduction [13], [15], [16], [40]. In this study, Hfq was also observed to upregulate the expression of OMPs, including that of Omp25 (BMEI1249), Omp25b (BMEI1007), Omp25c (BMEI1829), Omp31 (BMEII0844), OmpW (BMEI0454), BMEI0830, and BMEI1895. This dysregulation of OMPs could affect cell envelope stability in *B. melitensis* [41], thus improving bacterial resistance to cationic polypeptides such as polymyxin B (Fig. 2A).

**Figure 5:**
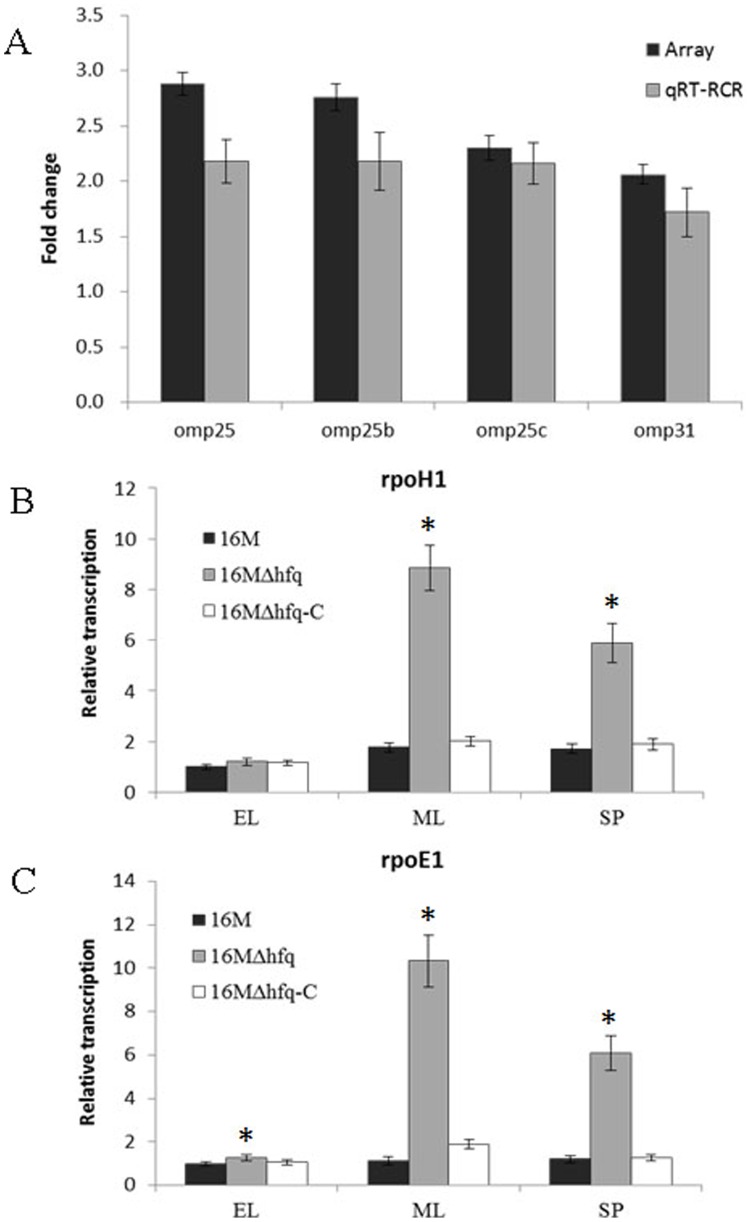
Hfq upregulated the expression of Omp25/Omp31 and the Sigma factors. A. Fold changes in the transcript abundances of *omp25*, *omp25b*, *omp25c*, and *omp31* genes were detected by microarray and qRT-PCR in 16 MΔhfq, relative to 16 M. B, C. Transcript abundances of *rpoH1* (B) and *rpoE1* (C) were detected in the 16 M, 16 MΔhfq, and 16 MΔhfq-C during early logarithmic (EL), mid-logarithmic (ML), and stationary phases (SP). Significant differences between the transcription abundances of *rpoH1* and *rpoE1* in the mutant and parent strain are indicated as follows: *, P<0.001.

Omp25 and Omp31 are 2 major *Brucella* OMPs. In *Brucella*, Omp25 is involved in membrane permeability in acidic medium [42] and has also been reported to be associated with virulence [43], [44]. Omp31 is one of the protective antigens of *Brucella* [45] and is also a hemin-binding protein involved in iron uptake [46]. To further confirm a negative regulatory role for Hfq in Omp25/Omp31 regulation, we determined the relative changes in *omp25*/*omp31* mRNA abundance by qRT-PCR. The results indicated that the transcript abundances of *omp25*, *omp25b*, *omp25c*, and *omp31* were elevated in the 16 MΔhfq mutant strain relative to those in the 16 M strain, which was consistent with the microarray analysis results (Fig. 5A). Although the regulation could be indirect, the fact that these OMPs are upregulated in the *hfq* mutant raises the possibility that OMPs biogenesis and outer membrane composition might be extensively regulated by sRNAs in *B. melitensis*, as reported in other systems [47]–[49].

### Sigma Factors

Alternative sigma factors contribute to bacterial resistance to environmental stress conditions and therefore contribute to the virulence of pathogenic bacteria. In *Salmonella typhimurium*, the involvement of Hfq in bacterial virulence was indicated by the requirement for derepression of RpoS translation [50]. *B. melitensis* does not possess an *rpoS*-like gene, but its genome contains genes that encode the other 6 sigma factors: RpoD (BMEI0532), RpoH1 (BMEI0280), RpoH2 (BMEI0378), RpoE1 (BMEI0371), RpoE2 (BMEII0072), and RpoN (BMEI1789) [51]. Among these 6 sigma factors, the transcript abundances of rpoH1 and rpoE1 were increased in the absence of Hfq. The rpoH1 gene encodes the σ^32^ homologues that mediate cytoplasmic heat shock responses. RpoH1 has been demonstrated to be involved in *B. melitensis* virulence [51]. The GroEL/S and DnaK/J chaperone proteins can regulate σ^32^ activity [52], [53]. Our microarray analysis revealed that GroEL/S was also upregulated in the hfq mutant cells. But whether Hfq regulates the σ^32^-mediated cytoplasmic heat shock response through GroEL/S remains to be elucidated.

In addition to RpoH1, the expression of the envelope stress sigma factor RpoE1 was also affected by the absence of Hfq. qRT-PCR analysis further confirmed that, in the Δhfq mutant, the rpoE1 transcript levels increased at different time points during the growth phase. Additionally, this increased σ^E^ level was restored to normal levels in the *hfq* complementary strain (Fig. 5C). These results indicated that Hfq exerted a negative effect on RpoE in *B. melitensis*, similar to reports for *E. coli* [54], [55], *S. typhimurium* [40], and *V. cholera* [13]. The σ^E^ stress response can be induced by misfolded envelope proteins, primarily the trimeric outer membrane porins [54]. In UPEC [15], *Salmonella* [40] and *Vibrio* [13], the overproduction of OMPs in Δhfq mutants might induce envelope stress and result in release of RpoE from the anti-sigma factor RseA thereby inducing σ^E^. The *B. melitensis* Δhfq strain also produces increased levels of OMPs and σ^E^, although no homologues of RseA were found in *Brucella*. Further studies are necessary to determine how Hfq regulates σ^E^ activity in *B. melitensis* and whether this regulation is OMP dependent.

### Oxidative, Acid and Heat Stress

Hfq was observed to modulate certain factors involved in adaptation to oxidative stress (SodC, AhpC, OsmC, and Dps), acid stress (HdeA, Omp25), and heat stress (GroEL, GroES, HtrA, and ClpP).

Previous reports have shown that oxidative killing is the primary mechanism employed by host phagocytes to control intracellular replication of the brucellae [56]. However, Brucellae are known to be able to withstand exposure to the lethal reactive oxygen intermediates produced by host phagocytes. Cu-Zn superoxide dismutase, which is encoded by the *sodC* gene (BMEII0581), is one of the primary antioxidants produced by *B. abortus* [18]. SodC protects *B. abortus* from O_2_ that is generated by oxidative bursts in host macrophages, and *B. abortus* SodC is essential to the establishment and maintenance of chronic infections. Interestingly, although the protein level of SodC was reduced in the absence of Hfq, the transcript abundance of *sodC* was increased in the Δhfq strain and decreased by the introduction of an *hfq*-complementation plasmid. This result indicated that *sodC* expression might be regulated by Hfq at multiple levels. Brucellae are exposed to potentially toxic levels of H_2_O_2_ both through the respiratory burst of host phagocytes and as a consequence of their aerobic metabolism [57], [58]. AhpC (BMEII0577) is the primary detoxifier of endogenous H_2_O_2_ that is generated by aerobic metabolism in *B. abortus* [58]. Phenotypic analysis revealed that the Δhfq mutant was extremely susceptible to H_2_O_2_ (Fig. 2A), this might be associated with the dysregulation of SodC and AhpC. Additionally, the osmotically inducible proteins OsmC (BMEII0409) and Dps (BMEI1980) were also notable. A Dps homolog in *E. coli* was reported to play a major role in the protection of bacteria from reactive oxygen intermediate (ROI) mediated damage during stationary-phase physiology [59]. An OsmC homolog was initially identified in *E. coli* as a protein that responds to osmotic stress [60]. In *E. coli*, OsmC participates in the defense against oxidative compounds, and its mutant exhibits reduced survival and increased sensitivity to oxidative stress. In mycobacteria, OsmC also plays important roles in peroxide metabolism and protection from oxidative stress [61]. However, in *B. melitensis*, the contributions of OsmC and Dps to virulence and survival in response to host macrophages need to be further defined.

During long-term residence in the phagosomal compartment of host macrophages, a major stress encountered by *Brucella* is exposure to acidic pH conditions. This low pH may represent an important environmental stimulus that triggers a genetic response required for successful adaptation to the intracellular environment. One well-known candidate for this response is the type IV secretion machinery encoded by the *virB* operon. Previously studies have shown that acidic pH in combination with nutrient deprivation induces expression of the *Brucella virB* operon [62], which is essential for the intracellular replication and virulence of *Brucella*. In our studies, we also found that the transcription level of *hfq* was highly induced under acidic nutrition deprivation condition (GEM 4.0). Interestingly, under nutrient rich conditions, the expression of *hfq* was lower in acidic medium (TSB 4.0) than that in neutral medium (TSB 7.0). The main reason is that the response of bacteria to low pH depends on the composition of the media [63], [64].

HdeA, a low pH-dependent chaperone, plays an important role in acid resistance in both *E. coli* [65] and *Shigella flexneri* [66]. Studies by Valderas and colleagues demonstrated that HdeA also contributes to acid resistance in *B. abortus* [19]. Our microarray analysis showed that *hdeA* exhibited modest repression (log2 ratio, 1.35) in the Δhfq strain. Further qRT-PCR analysis confirmed that the transcription level of *hdeA* was decreased when *hfq* was inactivated (Figure S2), indicating the negative role of Hfq on *hdeA*. Additionally, we found that the differences in abundance were more significantly under GEM 4.0 condition than TSB 7.0 condition (Figure S2). Valderas’ studies demonstrated that loss of *hdeA* did not impart the same degree of acid sensitivity upon the *B. abortus hdeA* mutant as that exhibited by the *hfq* mutant in the same assay [19]. This argues that inefficient expression of *hdeA* is not the sole basis for the remarkable acid sensitivity of *B. abortus hfq* mutant. Thus, brucellae might rely upon other genes or cellular components other than HdeA to resist the acidic conditions encountered in phagocytes. Omp25 (BMEI1249), which has been shown to be involved in the membrane permeability of *Brucella* in acidic medium [42], might also play a role in protecting *Brucella* from acidic pH exposures. Besides HdeA and Omp25, Hfq is certainly required for the expression of several other proteins that contribute to acid resistance in *Brucella*. Identification such proteins will help us to understand the role of Hfq in the intracellular survival of *Brucella*.

The GroELS chaperone machine is crucial in heat shock [67]. Results of transcriptomics and proteomics analyses both showed that GroEL (BMEII1048) was upregulated when *hfq* was inactivated. Increased GroEL expression was also documented in an *hfq* mutant of *Neisseria meningitidis* [68]. However, the *loss* of *hfq* resulted in the down-regulation of GroEL in *S. meliloti* [69], indicating that Hfq might regulate the expression of GroEL via different mechanisms in different bacteria. HtrA (BMEI0613) is generally thought to serve as a stress response protease in the periplasmic space, where it degrades proteins that are damaged by a variety of environmental stresses, including elevated temperatures and reactive oxygen intermediates. *Brucella* HtrA is important for adaption to the intracellular host macrophages environment [70]. The dysregulation of GroELS and HtrA correlates with the observation that the Δhfq mutant was obviously impaired with regard to high heat resistance (Fig. 2A).

### Flagellar Proteins

The bacterial flagellum is a complex organelle used for motility and thus helps bacteria to reach the most favorable environments. These complex organelles also play an important role in adhesion to substrates, biofilm formation, and virulence processes [71]. *Brucella* has long been described as non-motile. Nevertheless, homologues of flagellum-related proteins were described in *Brucella* [72]–[74]. *B. melitensis* 16 M has been reported to express some of the key genes of the flagellar apparatus and to assemble a sheathed flagellum that is required for virulence in a mouse infection model [75]. Another report showed that *B. melitensis* needs flagella to complete a normal infectious cycle, and flagellar gene mutants could not establish chronic infections in mice [76]. All of these data indicate that *B. melitensis* flagellar proteins play important roles in the infection process despite the fact that they are no longer capable of mediating flagellar motility. In the *B. melitensis* genome, 36 genes encode flagellar and motor proteins, and most of these genes are distributed in 3 clusters on the small chromosome. Interestingly, the microarray results revealed reduced transcript expression of nearly all of these genes in the mutant (Table S4), indicating that Hfq positively regulates the expression of flagellar proteins. Since the flagellar apparatus plays a role in *B. melitensis* virulence, the reduced expression of flagellar genes might contribute to the inability of the *B. melitensis hfq* mutant to maintain prolonged survival and chronic infection. The down-regulation of flagellar genes has been also reported for *hfq* mutants of *Salmonella* [32] and the α-proteobacterium *S. meliloti* [77].

Flagellar protein expression is highly regulated by environmental factors and regulatory proteins [71]. Several transcriptional regulators that control flagellar gene expression have been identified. Previously published data show that RpoE1 represses flagellar synthesis and filament lengths in *B. melitensis* [78]. In the present study, the abundance of the *rpoE1* transcript was increased in the absence of Hfq, whereas flagellar gene expression was decreased, suggesting that Hfq might also govern flagellar gene expression indirectly in *B. melitensis* through its negative effects on sigma factor availability. The transcriptional regulators VjbR (BMEII1116) [79] and FtcR (BMEII0158) [75] have been reported to directly activate the expression of flagellar apparatus in *B. melitensis*. Our results showed that the abundance of *vjbR* and *ftcR* transcript levels was decreased (−6.31 and −1.57 folds, respectively) in Δhfq mutant relative to the levels in 16 M, indicating that Hfq positively regulates *vjbR* and *ftcR* expression. Since Hfq is also an activator of flagellar gene transcription, we hypothesized that the flagellar apparatus might be directly regulated by Hfq or mediated through an intermediate transcriptional regulator such as *vjbR* or *ftcR*. Studying how Hfq regulates the flagellar apparatus in *Brucella* will be useful for understanding the pathogenicity of this strain.

### Protein Synthesis

Hfq also appears to play a significant role in protein synthesis. Forty-five genes that encoded ribosomal proteins and translation factors (e.g. elongation factor, translation initiation factor, and ribosome-binding factor) exhibited marked increases in mRNA levels in the Δhfq mutant strain, compared to the wild-type strain (Table S2). Strangely, none of the associated proteins were identified in proteomics analysis, suggesting that Hfq may repress protein synthesis in mRNA levels.

## Summary

The RNA-binding protein Hfq has emerged as a global post-transcriptional regulator of bacterial gene expression that participates in numerous regulatory pathways. In this study, we have demonstrated that Hfq modulates the expression of a wide range of genes and thus regulates the intracellular survival and virulence of *B. melitensis*. In the absence of Hfq, *B. melitensis* had a decreased growth rate and reduced survival in response to environmental stresses, suggesting a role for Hfq in stress resistance. The ability of brucellae to survive and multiply in the hostile host macrophages environment is a key factor in the development of brucellosis. Our data confirmed that the loss of Hfq reduced the intracellular survival of *B. melitensis* in both macrophage-like cells and mice. In order to survive in hostile environments, *B. melitensis* must adapt to environmental changes and respond quickly by adjusting the expression of genes, particularly those associated with stress responses and metabolism. Here we combined transcriptome and proteome analyses to identify targets that are directly or indirectly regulated by Hfq at a genome-wide scale, and this is the first study to determine the full repertoire of Hfq-dependent genes in *Brucella*. The results showed that 11% of all genes were affected by *hfq* disruption, indicating that Hfq is a key regulator of *Brucella* gene expression. Many of these target genes or proteins were involved in virulence and essential stress adaptations for intracellular survival or replication. The direction of regulation for many of these targets such as outer membrane proteins, flagellar proteins, *rpoE*, *virB*, and *luxS* generally agreed with previous reports. Besides acting alone to regulate gene expression, the RNA chaperone Hfq is a key factor in sRNAs-mediated regulation. Previous reports have shown that half of all known sRNAs in *Escherichia* [80] and *Salmonella* [81] associate with Hfq. Little is known about sRNAs in *Brucella* except for *abcR1* and *abcR2*, which were recently reported by Caswell et al [82]. Our work reveals potential target genes for sRNAs regulation in *B. melitensis*. In conclusion, this study has increased the number of known Hfq targets in *B. melitensis* and enabled the identification of putative sRNA targets. Further studies will be necessary to determine the sRNAs involved in such regulatory events in *B. melitensis*.

## Supporting Information

Figure S1 Two-dimensional gel electrophoresis patterns of *B. melitensis* 16 M and 16 MΔhfq. 16 M and 16 MΔhfq were firstly cultured in TSB to the stationary phase and then transferred into GEM4.0 for 30 min. Protein extracts (1 mg) of each strain were focused with IPG strips and run on 12% SDS-PAGE gels. The gels were stained with Coomassie Brilliant Blue G-250 and subjected to 2-DE analyses. The gels of 16 M and 16 MΔhfq were scanned and compared with ImageMaster 2D software. The labeled protein spots were the ones whose expressions were changed over 2 folds. The numbers marked on this map correspond to the spots numbers listed in Table S3.
(TIF)

Figure S2 Transcript abundances of *hdeA* were detected in the 16 M and 16 MΔhfq under GEM 4.0 and TSB 7.0 conditions. Significant differences between the transcription abundances of *hdeA* in the mutant and parent strain are indicated as follows: *, P<0.05; **, P<0.001.
(TIF)

Table S1 Primers used in this study.
(DOCX)

Table S2 Differentially expressed transcripts in *B. melitensis* 16 M and 16 MΔhfq.
(DOC)

Table S3 Differentially expressed proteins in *B. melitensis* 16 M and 16 MΔhfq.
(DOC)

Table S4 Hfq-dependent changes in transcript abundances of flagellar genes in *B. melitensis* 16 M.
(DOC)
