# Supplementary figures and images for: Impact of Hfq on Global Gene Expression and Intracellular Survival in Brucella melitensis
Source: PLoS One. 2013 Aug 19;8(8):e71933. doi: 10.1371/journal.pone.0071933 (PMC3747064; doi:10.1371/journal.pone.0071933)

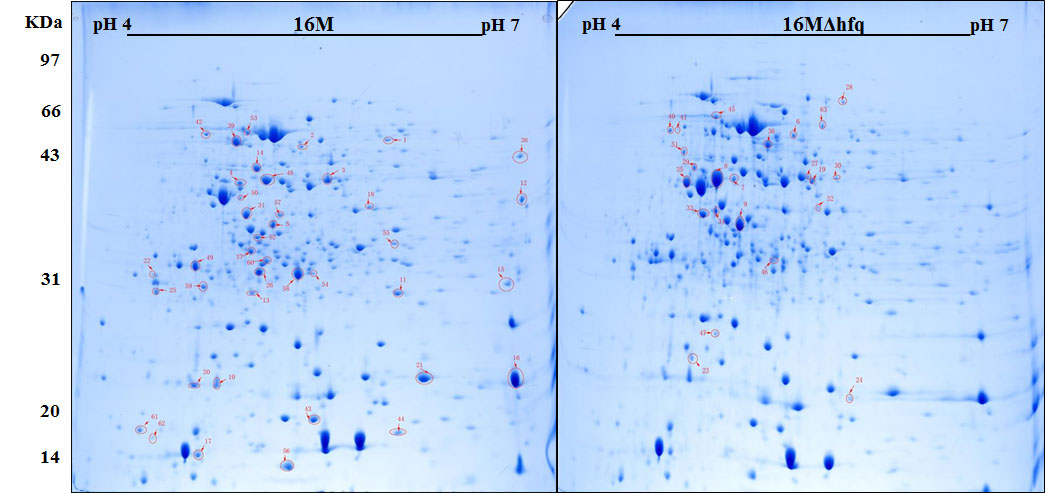

Supplement: Figure S1 — Two-dimensional gel electrophoresis patterns of B. melitensis 16 M and 16 MΔhfq. 16 M and 16 MΔhfq were firstly cultured in TSB to the stationary phase and then transferred into GEM4.0 for 30 min. Protein extracts (1 mg) of each strain were focused with IPG strips and run on 12% SDS-PAGE gels. The gels were stained with Coomassie Brilliant Blue G-250 and subjected to 2-DE analyses. The gels of 16 M and 16 MΔhfq were scanned and compared with ImageMaster 2D software. The labeled protein spots were the ones whose expressions were changed over 2 folds. The numbers marked on this map correspond to the spots numbers listed in Table S3. (TIF) [file pone.0071933.s001.tif]

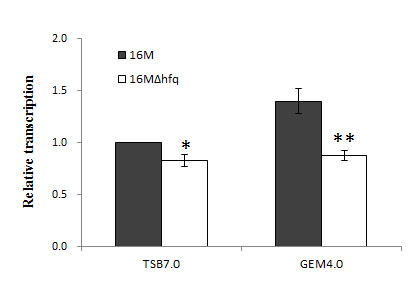

Supplement: Figure S2 — Transcript abundances of hdeA were detected in the 16 M and 16 MΔhfq under GEM 4.0 and TSB 7.0 conditions. Significant differences between the transcription abundances of hdeA in the mutant and parent strain are indicated as follows: *, P<0.05; **, P<0.001. (TIF) [file pone.0071933.s002.tif]
