# Supplementary material for: Impact of Hfq on Global Gene Expression and Intracellular Survival in Brucella melitensis
Source: PLoS One. 2013 Aug 19;8(8):e71933. doi: 10.1371/journal.pone.0071933 (PMC3747064; doi:10.1371/journal.pone.0071933)
Supplement: Table S1 — Primers used in this study. (DOCX) [file pone.0071933.s003.docx]

Table S1 Primers used in this study

| Primer Name | Primer sequence (5’-3’) |
| --- | --- |
| hfq-N-F | ACGTGGTACCTTGCTACGAGCCAATGAA |
| hfq-N-R | ACGTCTCGAGTCTGCTTGCGGACAGAAT |
| hfq-C-F | ACGTGTCGACGTTTGAAGGCGAGGAAGC |
| hfq -C-R | ACGTAAGCTTAGGACGGGAACAATGACG |
| pUC19K-F | ATCAGGACATAGCGTTGGC |
| hfq-I-R | CCATTCCTTTTCAAGATTGC |
| hfq-RT-F | GTCCGCAAGCAGAAGATT |
| hfq-RT-R | TTCCTCGCCTTCAAACAT |
| 16SrRNA-F | ACTAAGGGCGAGGGTTGC |
| 16srRNA-R | CACTGGACCATTACTGACGC |
| Omp25(BMEI1249) -RT- F | GAAGGTGATGCAGGTTATTCC |
| Omp25(BMEI1249)-RT- R | GTCGTCCAAGCCGTTGTT |
| Omp25b(BMEI1007) -RT- F | GTTGTTGCTCCGACCTTC |
| Omp25b(BMEI1007)-RT- R | ACCGTCTGGCTTTACTCG |
| Omp25c(BMEI1829) -RT- F | GACCGCTTCCTGCCCTAT |
| Omp25c(BMEI1829)-RT- R | CCGTAATCCTTATTGCCGTAG |
| Omp31(BMEII0844)-RT- F | TCGTCGGTGGTGTTCAGG |
| Omp31(BMEII0844)-RT- R | CGAGGTCGGTGTAGAGGTATT |
| RpoH1 (BMEI0280) -RT- F | GTTCTTCCACAGTTCCAGCCAATCG |
| RpoH1 (BMEI0280)-RT- R | AGCCCGACATAGCCCTCCT |
| RpoE1(BMEI0371)-RT-F | AACAGGTCCGTCGGTTGAG |
| RpoE1(BMEI0371)-RT-R | CGCAGAATGGTGAAGAGCC |
