# Supplementary material for: Impact of Hfq on Global Gene Expression and Intracellular Survival in Brucella melitensis
Source: PLoS One. 2013 Aug 19;8(8):e71933. doi: 10.1371/journal.pone.0071933 (PMC3747064; doi:10.1371/journal.pone.0071933)
Supplement: Table S2 — Differentially expressed transcripts in B. melitensis 16 M and 16 MΔhfq. (DOC) [file pone.0071933.s004.doc]

Table S2 Differentially expressed transcripts (>2-fold) in *B. melitensis* 16M and 16MΔhfq

| **Name** | **Fold change^a^** | **Gene** | **Putative function** |
| --- | --- | --- | --- |
| **Metabolism^b^** |  |  |  |
| Amino acid metabolism | |  |  |
| BMEI1759 | 2.74 | metH | B12-dependent methionine synthase |
| BMEI0577 | 2.30 | murD | UDP-N-acetylmuramoyl-L-alanyl-D-glutamate synthetase |
| BMEI2029 | 2.26 | - | S-adenosyl-L-homocysteine hydrolase |
| BMEI0930 | 2.22 | - | acetyltransferase |
| BMEI0931 | 2.18 | - | putative thiosulfate sulfurtransferase |
| BMEII0783 | 2.16 | - | Na(+)-linked D-alanine glycine permease |
| BMEII0356 | -2.01 | - | dihydroxy-acid dehydratase |
| BMEI0143 | -2.22 | - | threonine efflux protein |
| BMEII0059 | -2.23 | - | aminoquinate/shkimate dehydrogenase |
| BMEII0348 | -2.23 | - | 4-aminobutyrate aminotransferase |
| BMEI0103 | -2.70 | - | methionine gamma-lyase |
| BMEI1719 | -2.83 | - | sarcosine oxidase gamma subunit |
| BMEII0139 | -2.99 | ptpA | phosphotyrosyl phosphatase activator (PtpA) |
| BMEII0126 | -3.04 | - | amino acid permease |
| Carbohydrate metabolism | |  |  |
| BMEII0313 | -2.01 | - | glycerate dehydrogenase |
| BMEII0215 | -2.05 | - | 3-hydroxybutyryl-CoA dehydrogenase |
| BMEI2032 | -2.10 | - | PTS system, IIA component |
| BMEII0827 | -2.28 | - | glucose-1-phosphate cytidylyltransferase |
| BMEII0850 | -2.35 | - | GDP-fucose synthetase |
| BMEII0428 | -2.41 | - | D-erythrulose 4-phosphate dehydrogenase |
| BMEI1888 | -2.65 | - | lactoylglutathione lyase |
| BMEII0980 | -2.71 | - | ribitol 2-dehydrogenase |
| BMEII0387 | -2.87 | - | formyltetrahydrofolate deformylase |
| BMEI1511 | -2.88 | - | Phosphoglycerate mutase/fructose-2,6-bisphosphatase |
| BMEII0357 | -2.96 | - | 2-dehydro-3-deoxygalactonokinase |
| BMEII0355 | -3.03 | - | D-galactose 1-dehydrogenase |
| BMEII0571 | -4.07 | iolD | acetolactate synthase IolD |
| BMEII0572 | -4.56 | - | 5-dehydro-2-deoxygluconokinase |
| Energy metabolism | |  |  |
| BMEI1638 | 7.46 | - | putative oxidoreductase |
| BMEI0801 | 3.94 | pccB | propionyl-CoA carboxylase subunit beta |
| BMEI1545 | 3.66 | atpE | ATP synthase subunit C |
| BMEI0566 | 2.79 | - | soluble lytic murein transglycosylase |
| BMEI1088 | 2.59 | - | soluble lytic murein transglycosylase |
| BMEI0979 | 2.37 | - | glutamine synthetase |
| BMEI2020 | 2.29 | accD | acetyl-CoA carboxylase beta subunit |
| BMEI0251 | 2.11 | atpD | ATP synthase subunit B |
| BMEI0252 | 2.04 | atpC | ATP synthase subunit epsilon |
| BMEI0249 | 2.02 | atpA | ATP synthase subunit A |
| BMEII0974 | -2.15 | - | nitrous-oxide reductase |
| BMEII0988 | -2.27 | - | copper-containing nitrite reductase precursor |
| BMEII0907 | -2.47 | glsA | glutaminase |
| BMEI0615 | -2.58 | - | phosphoserine phosphatase |
| BMEII1001 | -3.06 | norE | nitric oxide reductase NorE protein |
| Inorganic ion metabolism | |  |  |
| BMEI1980 | 3.60 | dps | DNA protection during starvation conditions |
| BMEII0885 | 2.28 | - | 19 kDa periplasmic protein |
| BMEII0996 | -2.55 | norD | NorD protein |
| BMEII0110 | -3.02 | - | choline-sulfatase |
| Lipid metabolism | |  |  |
| BMEI0860 | 2.16 | - | Hypothetical Cytosolic Protein |
| BMEII0779 | -2.49 | fabH | 3-oxoacyl-(acyl carrier protein) synthase |
| Metabolism of cofactors and vitamins | | |  |
| BMEI0704 | -2.03 | - | uroporphyrin-III C-methyltransferase |
| BMEI1735 | -2.21 | thiG | thiazole synthase |
| BMEI1736 | -3.73 | thiE | thiamine-phosphate pyrophosphorylase |
| BMEI1768 | -2.07 | - | uroporphyrin-III C-methyltransferase |
| BMEI2039 | -2.20 | - | pantothenate kinase |
| BMEII0956 | -3.27 | - | 3-octaprenyl-4-hydroxybenzoate carboxy-lyase |
| Nucleotide metabolism | |  |  |
| BMEI1644 | 3.50 | - | dihydropyrimidinase |
| BMEI1643 | 3.31 | - | N-carbamoyl-L-amino acid amidohydrolase |
| BMEI1961 | 3.00 | pnp | polynucleotide phosphorylase |
| BMEI0781 | 2.63 | - | DNA-directed RNA polymerase alpha subunit |
| BMEI1639 | 2.54 | - | dihydropyrimidine dehydrogenase |
| BMEI0749 | 2.51 | - | DNA-directed RNA polymerase beta subunit |
| BMEI0750 | 2.31 | - | DNA-directed RNA polymerase beta' subunit |
| BMEI0778 | 2.11 | adk | adenylate kinase |
| BMEI0989 | -2.07 | - | thymidylate kinase |
| BMEI2056 | -2.29 | - | DNA polymerase III subunit epsilon |
| BMEI0296 | -2.37 | purE | phosphoribosylaminoimidazole carboxylase catalytic subunit |
| BMEI0295 | -2.49 | purK | phosphoribosylaminoimidazole carboxylase |
| Glycan biosynthesis and metabolism | | |  |
| BMEI0833 | 2.42 | lpxA | UDP-N-acetylglucosamine acyltransferase |
| BMEI0271 | -3.40 | mtgA | monofunctional biosynthetic peptidoglycan transglycosylase |
| Xenobiotics biodegradation and metabolism | | | |
| BMEII0643 | -2.28 | pcaI | 3-oxoadipate CoA-transferase subunit A |
| BMEII0636 | -2.57 | pcaH | protocatechuate 3,4-dioxygenase subunit beta |
| Other secondary metabolites | |  |  |
| BMEII0015 | 2.41 | - | homospermidine synthase |
| BMEII0016 | 2.14 | - | homospermidine synthase |
| **Membrane proteins** | |  |  |
| BMEI1249 | 2.88 | omp25 | 25 kDa outer-membrane immunogenic protein precursor |
| BMEI0830 | 2.78 | yaeT | outer membrane protein |
| BMEI1007 | 2.76 | omp25b | 25 kDa outer-membrane immunogenic protein precursor |
| BMEI1829 | 2.30 | omp25c | 25 kDa outer-membrane immunogenic protein precursor |
| BMEI0454 | 2.20 | ompW | outer membrane protein w precursor |
| BMEII0844 | 2.06 | omp31 | 31 kDa outer-membrane immunogenic protein precursor |
| BMEII0347 | -2.11 | - | membrane protein related to metalloendopeptidase |
| BMEII0832 | -2.14 | - | UDP-glucose 4-epimerase |
| BMEII0148 | -2.26 | - | serine protease |
| BMEI1872 | -2.27 | - | cell surface protein |
| BMEII0036 | -2.50 | - | outer membrane protein OprF |
| BMEII1069 | -2.63 | - | adhesin |
| BMEII0835 | -2.69 | - | glycosyl transferase |
| BMEII0380 | -2.86 | - | acriflavin resistance protein A |
| BMEI1342 | -3.00 | - | hypothetical protein |
| BMEI0359 | -3.44 | macA | periplasmic component of efflux system |
| **Transport** |  |  |  |
| Amino acid transport | |  |  |
| BMEI1211 | 6.93 | aapJ | general L-amino acid-binding periplasmic protein AapJ precursor |
| BMEI0258 | 6.71 | livH | branched-chain amino acid transport system permease protein LivH |
| BMEI0260 | 5.20 | livG | branched-chain amino acid transport ATP-binding protein BraF |
| BMEI0261 | 4.01 | livF | branched-chain amino acid transport ATP-binding protein BraG |
| BMEI0259 | 3.93 | livM | branched-chain amino acid transport system permease protein  LivM |
| BMEI1210 | 2.74 | aapQ | general L-amino acid transport system permease protein AapQ |
| BMEI0263 | 2.57 | livK | leucine-, isoleucine-, valine-, threonine-, and alanine-binding  protein precursor |
| BMEI1208 | 2.49 | aapP | general L-amino acid transport ATP-binding protein AapP |
| BMEI1930 | 2.12 | livK | leucine-, isoleucine-, valine-, threonine-, and alanine-binding  protein precursor |
| BMEII0099 | -2.12 | livG | branched-chain amino acid transport ATP-binding protein LivG |
| BMEII0632 | -2.23 | livH | branched-chain amino acid transport system permease protein LivH |
| BMEII0628 | -2.27 | livF | branched-chain amino acid transport ATP-binding protein LivF |
| BMEII0121 | -2.29 | livH | branched-chain amino acid transport system permease protein LivH |
| BMEII0100 | -2.43 | livG | branched-chain amino acid transport ATP-binding protein LivG |
| BMEII0119 | -2.70 | livF | branched-chain amino acid transport ATP-binding protein LivF |
| BMEI0112 | -3.21 | - | asparagine transport system permease protein |
| BMEI0111 | -4.55 | - | asparagine transport ATP-binding protein |
| Peptide transport | |  |  |
| BMEII0734 | 2.18 | oppA | periplasmic oligopeptide-binding protein precursor |
| BMEII0220 | -2.04 | oppB | oligopeptide transport system permease protein OppB |
| BMEII0209 | -2.13 | dppB | peptide ABC transporter permease DppB |
| BMEI0437 | -2.28 | dppD | peptide ABC transporter ATP-binding protein DppD |
| BMEII0859 | -2.77 | appA | oligopeptide-binding protein APPA precursor |
| BMEII0860 | -3.45 | appB | oligopeptide transport system permease protein AppB |
| Mineral and organic ion transport | | |  |
| BMEII0105 | 3.03 | - | iron-regulated outer membrane protein FrpB |
| BMEI0569 | 2.30 | mntH | putative manganese transport protein MntH |
| BMEII1120 | 2.30 | - | iron(III)-binding periplasmic protein precursor |
| BMEII0005 | 2.09 | modA | molybdate-binding periplasmic protein |
| BMEII0479 | -2.03 | - | ABC transporter substrate-binding protein |
| BMEII0108 | -2.14 | tauB | taurine transport ATP-binding protein TAUB |
| BMEII0604 | -2.17 | - | iron complex transport system ATP-binding protein |
| BMEI1840 | -2.21 | cysT | sulfate transport system permease protein cysT |
| BMEI1739 | -2.30 | thiX | ABC transporter membrane protein |
| BMEII0972 | -2.35 | nosD | copper-binding periplasmic protein precursor |
| BMEII0483 | -2.68 | - | ABC transporter membrane protein |
| BMEII0195 | -3.86 | attB | spermidine/putrescine ABC transporter permease |
| BMEI1727 | -4.41 | proV | glycine betaine/L-proline transport ATP-binding protein ProV |
| Saccharide and polyol transport | | |  |
| BMEII0590 | 2.31 | - | sugar-binding protein |
| BMEII0435 | 2.08 | rbsB | D-ribose-binding periplasmic protein precursor |
| BMEII0086 | -2.09 | mglC | galactoside transport system permease mglC |
| BMEII0360 | -2.21 | - | multiple sugar-binding periplasmic receptor ChvE precursor |
| BMEII0361 | -2.54 | - | sugar transport ATP-binding protein |
| BMEII0114 | -3.00 | ugpE | SN-glycerol-3-phosphate transport system permease protein UgpE |
| BMEII1053 | -3.14 | fucP | glucose/galactose transporter |
| Bacterial secretion system | |  |  |
| BMEII0275 | 2.19 | yidC | putative inner membrane protein translocase component YidC |
| BMEI0340 | 2.14 | - | peptidoglycan-associated lipoprotein |
| BMEI1084 | 2.12 | tatA | twin argininte translocase protein A |
| BMEII0030 | -2.03 | virB6 | channel protein VirB6-like protein |
| BMEII0033 | -2.66 | virB9 | channel protein VirB9-like protein |
| BMEII0028 | -3.16 | virB4 | ATPase VirB4-like protein |
| BMEII0035 | -4.73 | virB11 | ATPase VirB11-like protein |
| BMEII0027 | -5.89 | virB3 | VirB3 |
| Other transport systems | |  |  |
| BMEI0337 | 2.39 | - | biopolymer transport EXBD protein |
| BMEI0167 | -2.03 | amt | ammonium transporter |
| BMEII0851 | -2.07 | - | exopolysaccharide production protein EXOF precursor |
| BMEI2031 | -2.25 | - | phosphocarrier protein Hpr |
| BMEII0852 | -2.40 | - | succinoglycan biosynthesis transport protein exoP |
| BMEII0970 | -2.51 | - | membrane protein NOSY precursor |
| **Transcription** | |  |  |
| BMEI0371 | 3.13 | rpoE1 | RNA polymerase sigma-70 factor |
| BMEI1971 | 2.78 | - | transcriptional regulator |
| BMEI1510 | 2.69 | cspA | cold shock protein CSPA |
| BMEI1967 | 2.59 | nusA | transcription elongation factor NusA |
| BMEI0280 | 2.32 | rpoH1 | RNA polymerase sigma factor |
| BMEI0518 | 2.14 | cspA | cold shock protein CSPA |
| BMEI1194 | 2.08 | mucS | exopolysaccharide synthesis regulatory protein MUCS |
| BMEI1287 | -2.01 | rncS | ribonuclease III |
| BMEII0486 | -2.03 | nikR | nickel responsive regulator |
| BMEII0603 | -2.16 | - | hypothetical protein |
| BMEII0820 | -2.18 | - | ALS operon regulatory protein |
| BMEII0763 | -2.25 | - | ArsR family transcriptional regulator |
| BMEII0204 | -2.46 | - | GntR family transcriptional regulator |
| BMEII0804 | -3.57 | - | TETR family transcriptional regulator |
| BMEII0393 | -3.79 | - | ArsR family transcriptional regulator |
| BMEI1663 | -4.16 | - | antirepressor protein ANT |
| BMEII0219 | -5.58 | - | transcriptional regulator |
| **Translation** |  |  |  |
| BMEI0156 | 23.45 | rplS | 50S ribosomal protein L19 |
| BMEI0201 | 12.66 | rplU | LSU ribosomal protein L21P |
| BMEI0774 | 8.40 | rpsE | 30S ribosomal protein S5 |
| BMEI0775 | 6.03 | - | 50S ribosomal protein L30 |
| BMEI0754 | 3.60 | - | elongation factor EF-2 |
| BMEI0747 | 3.38 | rplJ | 50S ribosomal protein L10 |
| BMEI0753 | 3.21 | - | 30S ribosomal protein S7 |
| BMEI0757 | 3.18 | - | 50S ribosomal protein L3 |
| BMEI0772 | 3.11 | - | 50S ribosomal protein L6 |
| BMEI0934 | 3.09 | rhlE | ATP-dependent RNA helicase RHLE |
| BMEI0773 | 3.05 | rplR | 50S ribosomal protein L18 |
| BMEI0322 | 2.95 | - | 50S ribosomal protein L31 |
| BMEI0755 | 2.92 | tuf | elongation factor Tu |
| BMEI0769 | 2.90 | - | 50S ribosomal protein L5 |
| BMEI1965 | 2.90 | infB | translation initiation factor IF-2 |
| BMEI0771 | 2.88 | - | 30S ribosomal protein S8 |
| BMEI0742 | 2.83 | tuf | elongation factor Tu |
| BMEI0761 | 2.80 | rpsS | 30S ribosomal protein S19 |
| BMEI0758 | 2.64 | - | 50S ribosomal protein L4 |
| BMEI0748 | 2.61 | - | 50S ribosomal protein L7/L12 |
| BMEI1483 | 2.58 | - | 50S ribosomal protein L9 |
| BMEI0768 | 2.55 | rplX | 50S ribosomal protein L24 |
| BMEI0764 | 2.54 | rplP | 50S ribosomal protein L16 |
| BMEI2007 | 2.54 | rplT | 50S ribosomal protein L20 |
| BMEI0760 | 2.54 | rplB | 50S ribosomal protein L2 |
| BMEI1480 | 2.46 | rpsF | 30S ribosomal protein S6 |
| BMEI1962 | 2.43 | - | 30S ribosomal protein S15 |
| BMEI0780 | 2.37 | - | 30S ribosomal protein S11 |
| BMEI1915 | 2.35 | - | 30S ribosomal protein S1 |
| BMEI0756 | 2.35 | - | 30S ribosomal protein S10 |
| BMEI0770 | 2.33 | - | 30S ribosomal protein S14 |
| BMEI1963 | 2.33 | truB | tRNA pseudouridine synthase B |
| BMEI0762 | 2.29 | rplV | 50S ribosomal protein L22 |
| BMEI1481 | 2.20 | rpsR | 30S ribosomal protein S18 |
| BMEI0820 | 2.19 | - | translation initiation inhibitor |
| BMEI0759 | 2.14 | rplW | 50S ribosomal protein L23 |
| BMEI0481 | 2.14 | rplY | 50S ribosomal protein L25 |
| BMEI1964 | 2.14 | rbfA | ribosome-binding factor A |
| BMEI0746 | 2.13 | rplA | 50S ribosomal protein L1 |
| BMEI0776 | 2.10 | rplO | LSU ribosomal protein L15P |
| BMEI0824 | 2.05 | tsf | elongation factor Ts |
| BMEI1133 | 2.05 | - | 30S ribosomal protein S4 |
| BMEI1169 | 2.05 | - | 30S ribosomal protein S9 |
| BMEI0765 | 2.01 | - | LSU ribosomal protein L29P |
| BMEII0661 | 2.01 | - | 50S ribosomal protein L33 |
| BMEI0007 | -2.14 | gidA | glucose-inhibited division protein A |
| BMEI0429 | -2.15 | - | 23S ribosomal RNA methyltransferase |
| BMEI1064 | -2.17 | aat | leucyl/phenylalanyl-tRNA--protein transferase |
| BMEI1496 | -2.18 | rumA | tRNA (uracil-5-) -methyltransferase |
| BMEII0540 | -2.61 | - | regulator of purine biosynthesis |
| BMEI1103 | -3.10 | dusA | tRNA-dihydrouridine synthase A |
| **Cellular processes** | |  |  |
| Cell growth and death | |  |  |
| BMEI0585 | 3.68 | ftsZ | cell division protein FtsZ |
| BMEI0874 | 2.20 | clpP | ATP-dependent Clp protease proteolytic subunit |
| BMEI1444 | 2.05 | ccrM | adenine-specific methyltransferase |
| Cell motility |  |  |  |
| BMEI1692 | -2.00 | flgJ | flagellar protein FlgJ |
| BMEII1089 | -2.02 | flgB | flagellar basal body rod protein |
| BMEII0159 | -2.02 | flgE | flagellar hook protein |
| BMEII1107 | -2.08 | flgF | flagellar basal body rod protein FlgF |
| BMEII1080 | -2.10 | fliP | flagellar biosynthesis protein |
| BMEII0154 | -2.24 | motB | flagellar motor protein |
| BMEII1112 | -2.37 | fliN | flagellar motor switch protein FliN |
| BMEII0163 | -2.37 | flbG | flagellum biosynthesis repressor |
| BMEII0164 | -2.84 | flgD | flagellar basal body rod modification protein |
| BMEII1083 | -3.96 | motE | motor |
| BMEII0155 | -7.16 | motC | chemotaxis protein |
| Other cellular processes | |  |  |
| BMEII0581 | 2.55 | sodC | superoxide dismutase (Cu-Zn) |
| **Signal transduction** | |  |  |
| BMEI0374 | 2.74 | - | sensory transduction histidine kinase |
| BMEI0423 | 2.04 | ctrA | response regulator CtrA |
| BMEII0660 | -2.07 | pleD | response regulator PleD |
| BMEII0904 | -2.13 | - | diguanylate phosphodiesterase |
| BMEII0854 | -2.17 | - | CRP family transcriptional regulator |
| BMEII1009 | -2.33 | - | c-di-GMP phosphodiesterase A |
| BMEI1903 | -2.63 | cyc | Cytochrome C-552 |
| BMEI1448 | -2.65 | - | c-di-GMP phosphodiesterase A-related protein |
| BMEI1648 | -2.66 | - | Sensory Transduction Protein Kinase |
| BMEI1863 | -2.95 | - | low molecular weight phosphotyrosine protein phosphatase |
| BMEII1116 | -6.31 | vjbR | LuxR family transcriptional regulator |
| **Posttranslational modification, chaperones** | | | |
| BMEII1047 | 3.54 | groES | co-chaperonin GroES |
| BMEII0577 | 3.41 | ahpC | alkyl hydroperoxide reductase C22 protein |
| BMEII1048 | 2.00 | groEL | chaperonin GroEL |
| BMEI1333 | -2.05 | ccmE | cytochrome c-type biogenesis protein CcmE |
| BMEI1327 | -2.79 | glnE | glutamate-ammonia-ligase adenylyltransferase |
| **Replication, recombination and repair** | | | |
| BMEI1402 | 2.02 | - | transposase |
| BMEI1406 | -2.06 | - | transposase |
| BMEI2023 | -2.13 | - | ATP-dependent nuclease subunit A |
| BMEII0739 | -2.24 | alkB | alkylated DNA repair protein AlkB |
| BMEI0126 | -2.33 | mutT | mutator protein MutT |
| BMEI2024 | -2.45 | - | Hypothetical Cytosolic Protein |
| BMEI1661 | -2.45 | - | recombinase |
| BMEII0228 | -2.71 | - | transposase |
| BMEI0215 | -2.74 | nudH | dinucleoside polyphosphate hydrolase |
| BMEI0902 | -2.79 | - | recombinase |
| **General function prediction only** | | |  |
| BMEI0804 | 5.54 | - | Hypothetical Cytosolic Protein |
| BMEI0873 | 3.07 | hflX | GTP-binding protein HflX |
| BMEII0997 | -2.03 | norQ | NorQ protein |
| BMEI0319 | -2.05 | bioY | BioY protein |
| BMEII0129 | -2.15 | - | hydrolase |
| BMEI0136 | -2.18 | - | ATPase n2B |
| BMEII0611 | -3.06 | - | hypothetical protein |
| BMEI0383 | -3.29 | - | 5-methylcytosine-specific restriction protein A |
| **Unknown function and hypothetical protein** | | | |
| BMEI0372 | 9.79 | - | two-component response regulator |
| BMEII0552 | 6.30 | - | hypothetical protein |
| BMEI0668 | 5.31 | - | calcium binding protein |
| BMEI0632 | 5.15 | - | hypothetical membrane spanning protein |
| BMEI0373 | 4.38 | - | hypothetical protein |
| BMEI1305 | 4.35 | - | porin |
| BMEI1306 | 4.05 | - | porin |
| BMEII0054 | 3.78 | - | hypothetical protein |
| BMEI1866 | 3.59 | - | hypothetical protein |
| BMEI0369 | 3.35 | - | hypothetical protein |
| BMEI0803 | 3.28 | - | hypothetical protein |
| BMEI1741 | 3.19 | - | hypothetical protein |
| BMEI0262 | 3.12 | - | hypothetical protein |
| BMEI1509 | 3.01 | - | hypothetical protein |
| BMEI1846 | 2.96 | - | response regulator receiver protein ExsF |
| BMEI1215 | 2.94 | - | hypothetical membrane spanning protein |
| BMEI0193 | 2.86 | - | hypothetical protein |
| BMEI0721 | 2.75 | - | hypothetical cytosolic protein |
| BMEII0501 | 2.71 | - | hypothetical cytosolic protein |
| BMEII0733 | 2.68 | - | hypothetical protein |
| BMEI1364 | 2.57 | - | transcriptional regulatory protein MucR |
| BMEI0805 | 2.56 | - | hypothetical protein |
| BMEI0299 | 2.44 | - | hypothetical protein |
| BMEI1242 | 2.44 | - | hypothetical membrane spanning protein |
| BMEI1514 | 2.40 | - | hypothetical protein |
| BMEI1474 | 2.39 | - | hypothetical protein |
| BMEI1584 | 2.38 | - | invasion protein B |
| BMEI0620 | 2.36 | - | hypothetical protein |
| BMEI1072 | 2.34 | - | hypothetical protein |
| BMEI0368 | 2.33 | - | hypothetical protein |
| BMEI1508 | 2.30 | - | putative lipoprotein |
| BMEII1102 | 2.21 | - | hypothetical protein |
| BMEI0186 | 2.20 | - | hypothetical cytosolic protein |
| BMEI0497 | 2.15 | - | hypothetical membrane spanning protein |
| BMEI1004 | 2.14 | - | hypothetical protein |
| BMEI1201 | 2.08 | - | hypothetical cytosolic protein |
| BMEII0652 | 2.07 | - | hypothetical protein |
| BMEI1932 | 2.07 | - | hypothetical cytosolic protein |
| BMEI1476 | 2.06 | - | hypothetical protein |
| BMEI1162 | 2.06 | - | hypothetical protein |
| BMEI1417 | 2.05 | - | perosamine synthetase WbkB |
| BMEI1637 | 2.05 | - | CoxG protein |
| BMEII1067 | 2.02 | - | hypothetical cytosolic protein |
| BMEI1515 | 2.01 | - | hypothetical protein |
| BMEI0289 | 2.00 | - | hypothetical cytosolic protein |
| BMEI1312 | -2.00 | - | hypothetical membrane spanning protein |
| BMEI1341 | -2.02 | - | phage host specificity protein |
| BMEI1660 | -2.04 | - | hypothetical protein |
| BMEII0811 | -2.06 | - | hypothetical membrane spanning protein |
| BMEI1697 | -2.06 | - | virulence-associated protein E |
| BMEI0031 | -2.08 | - | hypothetical cytosolic protein |
| BMEI0720 | -2.11 | - | sugar fermentation stimulation protein |
| BMEI1696 | -2.13 | - | hypothetical membrane spanning protein |
| BMEII1052 | -2.16 | - | transporter |
| BMEI0951 | -2.17 | - | amino acid regulated cytosolic protein |
| BMEI1896 | -2.17 | - | hypothetical membrane spanning protein |
| BMEII0715 | -2.19 | - | preprotein translocase subunit-like protein |
| BMEII0169 | -2.22 | - | hypothetical protein |
| BMEII0539 | -2.23 | - | hypothetical cytosolic protein |
| BMEII0795 | -2.27 | - | multidrug resistance protein B |
| BMEII1071 | -2.28 | - | hypothetical protein |
| BMEII0987 | -2.29 | - | NirV precursor |
| BMEI1677 | -2.30 | - | hypothetical protein |
| BMEII0538 | -2.31 | - | hypothetical protein |
| BMEI0879 | -2.31 | - | hypothetical protein |
| BMEI0487 | -2.34 | - | ATP synthase beta subunit/transription termination factor rho |
| BMEI1344 | -2.35 | - | hypothetical protein |
| BMEI1384 | -2.42 | - | AraC family transcriptional regulator |
| BMEII0315 | -2.45 | - | hypothetical protein |
| BMEII0379 | -2.47 | - | hypothetical protein |
| BMEII0045 | -2.51 | - | hypothetical protein |
| BMEI1678 | -2.56 | - | hypothetical protein |
| BMEI1662 | -2.57 | - | hypothetical protein |
| BMEII0547 | -2.58 | - | hypothetical Cytosolic Protein |
| BMEI0119 | -2.58 | - | hypothetical protein |
| BMEII0995 | -2.63 | - | hypothetical protein |
| BMEII1012 | -2.64 | - | hypothetical protein |
| BMEI1520 | -2.64 | - | response regulator protein |
| BMEII0128 | -2.66 | - | hypothetical protein |
| BMEI1656 | -2.76 | - | hypothetical protein |
| BMEII0803 | -2.78 | - | multidrug resistance protein A |
| BMEII0188 | -2.79 | - | hypothetical cytosolic protein |
| BMEII0191 | -2.83 | - | hypothetical protein |
| BMEII0664 | -2.83 | - | hypothetical protein |
| BMEII0331 | -2.86 | - | hypothetical cytosolic protein |
| BMEI0151 | -2.88 | - | hypothetical protein |
| BMEI1319 | -2.88 | - | hypothetical membrane spanning protein |
| BMEI1871 | -2.90 | - | hypothetical protein |
| BMEII0955 | -2.91 | - | hypothetical protein |
| BMEII0187 | -3.09 | - | hypothetical cytosolic protein |
| BMEI1695 | -3.18 | - | hypothetical protein |
| BMEI1280 | -3.18 | - | hypothetical Ccytosolic protein |
| BMEI1686 | -3.49 | - | hypothetical protein |
| BMEII0243 | -3.65 | - | hypothetical protein |
| BMEII1115 | -3.84 | - | hypothetical protein |
| BMEI0904 | -5.74 | - | hypothetical protein |

a: Fold change represents the mRNA abundance in 16MΔhfq mutant compared with that in the 16M. Positive numbers represent increases, while negative numbers represent decreases.

b: Functional classification according to KEGG ( <http://www.genome.jp/kegg/>) and *B. melitensis* 16M genome sequence annotation (NC_003317 and NC_003318).
