# Supplementary material for: Impact of Hfq on Global Gene Expression and Intracellular Survival in Brucella melitensis
Source: PLoS One. 2013 Aug 19;8(8):e71933. doi: 10.1371/journal.pone.0071933 (PMC3747064; doi:10.1371/journal.pone.0071933)
Supplement: Table S3 — Differentially expressed proteins in B. melitensis 16 M and 16 MΔhfq. (DOC) [file pone.0071933.s005.doc]

Table S3 Differentially expressed proteins (>2-fold) in *B. melitensis* 16M and 16MΔhfq

| **Locus** | **Protein description** | **Protein** | **Spot No.** | **Regulation^a^** |
| --- | --- | --- | --- | --- |
| **Metabolism^b^** |  |  |  |  |
| Amino acid metabolism | |  |  |  |
| BMEI0574 | UDP-N-acetylmuramoylalanyl-D-glutamate—  2,6-diaminopimelateligase | MurE | 1 | down |
| BMEI1668 | histidinol dehydrogenase | HisD | 2 | down |
| Carbohydrate metabolism | |  |  |  |
| BMEI0244 | translaldolase | - | 11 | down |
| BMEI0310 | glyceraldehyde-3-phosphate dehydrogenase | GapA | 12 | down |
| BMEI0496 | hydroxyacylglutathione hydrolase | - | 13 | down |
| BMEI0851 | phosphopyruvate hydratase | Eno | 14 | down |
| BMEII0248 | phosphoglyceromutase | - | 15 | down |
| Energy metabolism | |  |  |  |
| BMEI0252^#^ | ATP synthase F0F1 subunit epsilon | AtpC | 17 | down |
| BMEI0933 | cysteine synthase A | CysK | 18 | down |
| BMEII0559 | glycine cleavage system aminomethyltransferase T | GcvT | 19 | up |
| Inorganic ion metabolism | |  |  |  |
| BMEII0704 | bacterioferritin | - | 20 | down |
| BMEII0885^#^ | 19 kDa periplasmic protein | - | 21 | down |
| Lipid metabolism | |  |  |  |
| BMEI0032 | 3-oxoacyl-ACP reductase | - | 22 | down |
| BMEI1062 | acetyl-CoA carboxylase biotin carboxyl carrier protein subunit | - | 23 | up |
| BMEI1923 | isovaleryl-CoA dehydrogenase | - | 3 | down |
| BMEI1956 | 3-hydroxydecanoyl-ACP dehydratase | - | 24 | up |
| Metabolism of cofactors and vitamins | |  |  |  |
| BMEI0694 | COBW protein | - | 29 | up |
| BMEI1505 | GTP cyclohydrolase II | - | 30 | up |
| Other metabolism | |  |  |  |
| BMEI1643^#^ | allantoate amidohydrolase | - | 36 | up |
| BMEI1708 | 2-hydroxyhepta-2,4-diene-1,7-dioate isomerase | - | 37 | down |
| **Membrane proteins** | |  |  |  |
| BMEI0421 | pleiotropic regulatory protein | - | 27 | up |
| BMEI1895 | outer membrane protein | - | 28 | up |
| **Transport** |  |  |  |  |
| Amino acid transport | |  |  |  |
| BMEI1930^#^ | leucine-, isoleucine-, valine-, threonine-, and alanine-binding  protein precursor | LivK | 5 | down |
| BMEII0487 | nickel-binding periplasmic protein precursor | - | 6 | down |
| BMEI0263^#^ | leucine-, isoleucine-, valine-, threonine-, and alanine-binding  protein precursor | LivK | 4, 7, 8 | up |
| BMEI1211^#^ | general L-amino acid-binding periplasmic protein AapJ precursor | AapJ | 9 | up |
| Peptide transport | |  |  |  |
| BMEII0735 | periplasmic oligopeptide-binding protein precursor | OppA | 39 | down |
| BMEII0203 | binding protein YddS precursor | YddS | 40, 41 | up |
| Mineral and organic ion transport | |  |  |  |
| BMEII0584 | iron(III)-binding periplasmic protein precursor | - | 31 | down |
| BMEII0535 | iron(III) dicitrate-binding periplasmic protein | - | 32 | up |
| BMEII0923 | spermidine/putrescine-binding periplasmic protein | - | 33, 34 | up |
| BMEII1120^#^ | iron(III)-binding periplasmic protein precursor | - | 35 | up |
| Saccharide and polyol transport | |  |  |  |
| BMEI1716 | trehalose/maltose binding protein | - | 48 | down |
| BMEII0435^#^ | D-ribose-binding periplasmic protein precursor | RbsB | 49 | down |
| BMEII0590^#^ | sugar-binding protein | - | 50 | down |
| BMEII0945 | maltose-binding periplasmic protein | ThuE | 51 | up |
| Bacterial secretion system | |  |  |  |
| BMEI2055 | preprotein translocase subunit SecB | SecB | 10 | down |
| Other transport systems | |  |  |  |
| BMEI0339 | translocation protein TolB | TolB | 38 | down |
| **Translation** |  |  |  |  |
| BMEI1068 | aspartyl/glutamyl-tRNA amidotransferase subunit B | GatB | 53 | down |
| BMEI1787 | sigma54 modulation protein / SSU ribosomal protein S30P | - | 54 | down |
| **Cellular processes** | |  |  |  |
| BMEII0581^#^ | superoxide dismutase (Cu-Zn) | SodC | 16 | down |
| **Signal transduction** | |  |  |  |
| BMEI1989 | phosphate-binding periplasmic protein | PstS | 52 | down |
| **Posttranslational modification, chaperones** | |  |  |  |
| BMEI0613 | protease do | HtrA | 42 | down |
| BMEI1456 | thiol peroxidase | - | 43 | down |
| BMEII0409 | osmotically inducible protein C | OsmC | 44 | down |
| BMEI1069 | trigger factor | Tif | 45 | up |
| BMEII1048^#^ | chaperonin GroEL | GroEL | 46, 47 | up |
| **Unknown function and hypothetical protein** | |  |  |  |
| BMEI0290 | hypothetical protein BMEI0290 | - | 55 | down |
| BMEI0368^#^ | hypothetical protein BMEI0368 | - | 56 | down |
| BMEI0507 | hypothetical protein BMEI0507 | - | 57 | down |
| BMEI0536 | periplasmic immunogenic protein | Bp26 | 58 | down |
| BMEI1033 | hypothetical protein BMEI1033 | - | 59 | down |
| BMEI1092 | hypothetical protein BMEI1092 | - | 60 | down |
| BMEI1843 | hypothetical protein BMEI1843 | - | 61, 62 | down |
| BMEI1827 | hypothetical protein BMEI1827 | - | 63 | up |

a: Proteins upshifted in the 16MΔhfq mutant are marked with ‘‘up ’’, and those downshifted with ‘‘down’’;

b: Functional classification according to KEGG ( <http://www.genome.jp/kegg/>) and *B. melitensis* 16M genome sequence annotation (NC_003317 and NC_003318).

#: The corresponding coding transcripts were also found to be up/downregulated in the 16MΔhfq mutant.
