# Supplementary material for: Impact of Hfq on Global Gene Expression and Intracellular Survival in Brucella melitensis
Source: PLoS One. 2013 Aug 19;8(8):e71933. doi: 10.1371/journal.pone.0071933 (PMC3747064; doi:10.1371/journal.pone.0071933)
Supplement: Table S4 — Hfq-dependent changes in transcript abundances of flagellar genes in B. melitensis 16 M. (DOC) [file pone.0071933.s006.doc]

Table S4 Hfq-dependent changes in transcript abundances of flagellar proteins in *B. melitensis* 16M

| **Lous** | **Predicted function** | **Gene^a^** | **Fold change^b^** |
| --- | --- | --- | --- |
| BMEII0150 | flagellin | fliC | -1.55 |
| BMEII0151 | flagellar M-ring protein FliF | fliF | -1.20 |
| BMEII0152 | flagellar M-ring protein FliF | fliF | -1.95 |
| BMEII0154^#^ | flagellar motor protein MotB | motB | -2.24 |
| BMEII0155^#^ | motor | motC | -7.16 |
| BMEII0156 | motor | motD | -1.71 |
| BMEII0159^#^ | flagellar hook protein FlgE | flgE | -2.02 |
| BMEII0160 | flagellar hook-associated protein FlgK | flgK | -1.89 |
| BMEII0161 | flagellar hook-associated protein FlgL | flgL | -1.31 |
| BMEII0162 | flagellar biosynthesis regulatory protein FlaF | flaF | -1.22 |
| BMEII0163^#^ | flagellar biosynthesis repressor FlbT | flbT | -2.37 |
| BMEII0164^#^ | flagellar basal body rod modification protein | flgD | -2.84 |
| BMEII0165 | flagellar biosynthesis protein FliQ | fliQ | ND |
| BMEII0166 | flagellar biosynthetic protein FlhA | flhA | -1.67 |
| BMEII0167 | flagellar biosynthesis protein | flhA | -1.53 |
| BMEII0168 | flagellar biosynthesis protein FliR | fliR | -1.21 |
| BMEII0170 | flagellar protein FLGJ | flgJ | -1.53 |
| BMEII1080^#^ | flagellar biosynthesis protein FliP | fliP | -2.10 |
| BMEII1081 | flagellar protein FliL | fliL | -1.85 |
| BMEII1082 | flagellar basal body L-ring protein | flgH | -1.71 |
| BMEII1083^#^ | motor | motE | -3.96 |
| BMEII1084 | flagellar basal body P-ring protein | flgL | -1.68 |
| BMEII1085 | flagellar basal body P-ring biosynthesis protein FlgA | flgA | -1.84 |
| BMEII1086 | flagellar basal body rod protein FlgG | flgG | -1.12 |
| BMEII1087 | flagellar hook-basal body protein FliE | fliE | -1.69 |
| BMEII1088 | flagellar basal body rod protein FlgC | flgC | -1.25 |
| BMEII1089^#^ | flagellar basal body rod protein FlgB | flgF | -2.02 |
| BMEII1105 | flagellum ATP synthase | fliL | -1.43 |
| BMEII1107^#^ | flagellar basal-body rod protein FlgF | flgF | -2.08 |
| BMEII1108 | flagellar basal-body rod protein FlgF | flgF | -1.44 |
| BMEII1109 | flagellar motor protein MotA | motA | -1.16 |
| BMEII1110 | flagellar motor switch protein FliM | fliM | -1.12 |
| BMEII1112^#^ | flagellar motor switch protein FliN | fliN | -2.37 |
| BMEII1113 | flagellar motor switch protein FliG | fliG | -1.32 |
| BMEII1114 | flagellar biosynthesis protein FlhB | flhB | -1.86 |
| BMEI1692^#^ | flagellar protein FlgJ | flgJ | -2.00 |

a: In agreement with the name given for flagellar genes of *E. coli*, *Bacillus subtilis*, or *S. meliloti*;

b: Fold change represents the mRNA abundance in 16MΔhfq mutant compared with that in the 16M. Negative numbers represent decreases;

ND: Undetectable due to low abundance of mRNA or low efficiency of the corresponding primer pairs;

#: The fold change >2.
